# Synthesis and biological evaluation of ursolic acid derivatives bearing triazole moieties as potential anti-*Toxoplasma gondii* agents

**DOI:** 10.1080/14756366.2019.1584622

**Published:** 2019-03-05

**Authors:** Tian Luan, Chunmei Jin, Chun-Mei Jin, Guo-Hua Gong, Zhe-Shan Quan

**Affiliations:** a Key Laboratory of Natural Resources and Functional Molecules of the Changbai Mountain, Affiliated Ministry of Education, College of Pharmacy, Yanbian University, Yanji, Jilin, China;; b First Clinical Medical College of Inner Mongolia University for Nationalities, Tongliao, China;; c Inner Mongolia Key Laboratory of Mongolian Medicine Pharmacology for Cardio-Cerebral Vascular System, Inner Mongolia University for Nationalities, Tongliao, China

**Keywords:** *Toxoplasma gondii*, ursolic acid, molecular docking, TgCDPK1, *in vivo*, *in vitro*

## Abstract

Ursolic acid (**UA**), a plant-derived compound, has many properties beneficial to health. In the present study, we synthesised three series of novel **UA** derivatives and evaluated their anti-*Toxoplasma gondii* activity both *in vitro* and *in vivo*. Most derivatives exhibited an improved anti-*T. gondii* activity *in vitro* when compared with **UA** (parent compound), whereas compound **3d** exhibited the most potent anti-*T. gondii* activity *in vivo*. Spiramycin served as the positive control. Additionally, determination of biochemical parameters, including the liver and spleen indexes, indicated compound **3d** to effectively reduce hepatotoxicity and significantly enhance anti-oxidative effects, as compared with **UA**. Furthermore, our molecular docking study indicated compound **3d** to possess a strong binding affinity for *T. gondii* calcium-dependent protein kinase 1 (TgCDPK1). Based on these findings, we conclude that compound **3d**, a derivative of **UA**, could act as a potential inhibitor of TgCDPK1.

## 1. Introduction

*Toxoplasma gondii* is an opportunistic pathogen that causes infection in human beings and various animals, thereby severely impairing their health. Congenital toxoplasmosis, caused by *T. gondii*, is especially harmful to pregnant women as the infection may result in abortion, stillbirth, and abnormality of foetus thinking barrier. The infection could also be fatal for immuno-compromised patients^1^. Owing to the complexity of *T. gondii* life cycle, its multifarious pathogenesis and different biological characteristics, no preventive and medicine-specific treatment exists currently. Traditional anti-*T. gondii* drugs have various disadvantages, such as the inability to completely kill the protozoa and oocysts, high toxicity, frequent recurrence, and failure in immuno-compromised individuals^2^^,^^3^. Considering the increasing percentage of natural product-based drugs in the market in the past years, researchers have now focussed their attention to plant-based compounds with anti-*T. gondii* activity. Moreover, several studies have shown natural products and their derivatives to exert strong anti-*T. gondii* effects, making these an attractive source of anti-*T. gondii* drugs^4^^,^^5^. In this regard, structural modifications of natural products to generate effective and less-toxic derivatives are considered to be very promising for the development of anti-*T. gondii* drugs.

Pentacyclic triterpenes are a diverse and large class of natural products that are widely distributed in the plant kingdom. Over the decades, the synthesis of novel pentacyclic triterpenes has gained much attention in medicinal chemistry. Among these, ursolic acid (**UA**) and its derivatives have been reported to possess a wide range of biological activities, including anti-cancer^6^^,^^7^, anti-diabetic^8^, anti-HIV^9^, anti-malarial^10^, anti-microbial, and anti-inflammatory activities^11^^,^^12^. Until recently, Choi et al. reported that **UA** not only has strong anti-proliferative activity against *T. gondii* activity as well as increases survival of *T. gondii*-infected mice but also has the potential to be used as a promising anti-*T. gondii* candidate for developing effective anti-parasitic drugs^13^. To the best of our knowledge, studies related to anti-*T. gondii* activity of any **UA** derivatives have not yet been reported. Besides, the higher cytotoxicity *in vitro* and the low bioavailability *in vivo* of **UA** restrict its clinical application^14^^,^^15^. Therefore, the present study involved synthesis of different structurally modified compounds of **UA** with significantly improved anti-*T. gondii* activity and lower toxicity.

**Figure 1.**
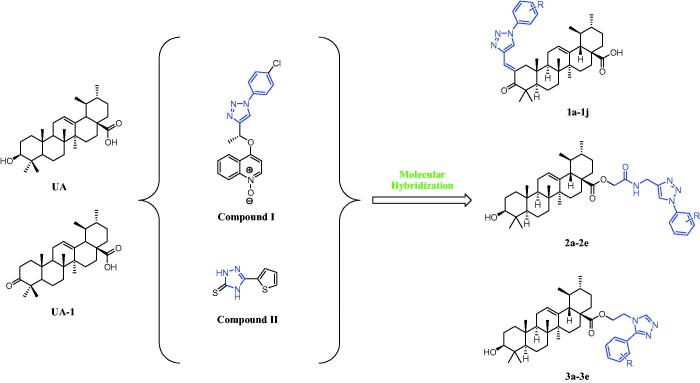
Design of target compounds based on the combination principles.

Recently, the chemistry of triazoles and their fused phenyl derivatives has received considerable attention owing to their effective biological and synthetic importance^16–18^. Sharling et al. reported that a series of 1,2,3-triazoles conjugate phenyl derivatives facilitated the development of potential anti-parasitic agents, of which, five derivatives exhibited excellent *in vitro* selectivity for *T. gondii*. Among these, compound **1** (Figure 1) exhibited the most potent anti-*T. gondii* activity with a selectivity value of more than 120^18^. Furthermore, Dzitko et al. reported anti-*T. gondii* activity of 3-(thiophen-2-yl)-1,2,4-triazole-5-thione (compound **2**). The compound displayed significant and reproducible anti-parasitic effects *in vitro*, with selectivity values of 4.58 and 5.21 using ^3^[H]uracil incorporation method and qRT-PCR, respectively^19^. These studies indicate triazole-based compounds to have potential inhibitory activity against anti-*T. gondii*.

The aforementioned findings stimulated our interest in designing and synthesising three series of novel **UA** derivatives by linking different fragments containing 1,2,3-triazole and 1,2,4-triazole and studying their effects against *T. gondii*, initially at the cellular level. We next tested each of these derivatives for the strongest anti-*T. gondii* activity *in vivo*, since *in vivo* effects are an important factor in evaluating anti-parasitic activity. Finally, we aimed to gain a better understanding of the molecular basis of inhibitory potency of compounds against *T. gondii.* For this, we identified three enzymes through literature search as reasonable targets for discovering anti-*T. gondii* agents, and by using the molecular docking approach, we aimed at finding the possible target.

## 2. Materials and methods

### 2.1. General procedures

All reactions were monitored by thin-layer chromatography (TLC) performed on silica gel plates. Melting points were determined in open capillary tubes and were uncorrected. Purity of final products was determined using a preparative high-performance liquid chromatography (HPLC) system (HP-Q-P050; Agela Technologies) with a C-18 column as the stationary phase (Agela Technologies, Venusil PrepG, 120 Å, 10 μm, 10 mm × 250 mm). The nuclear magnetic resonance (^1^H-NMR and ^13^C-NMR) spectra were recorded with AV-300 spectrometers (Bruker BioSpin, Switzerland); all chemical shifts were expressed in ppm relative to tetramethylsilane (TMS), used as the internal standard. High-resolution mass spectra were recorded using the Thermo Scientific LTQ Orbitrap XL in the electrospray ionisation (ESI) mode. Major chemicals were purchased from Aldrich Chemical Corporation (Milwaukee, WI, USA). All other chemicals were of analytical grade.

### 2.2. General procedure for synthesis of intermediates (**UA-1, Ia-Ij, IIa-IIe, IIIa-IIIe**)

**Scheme 1.**
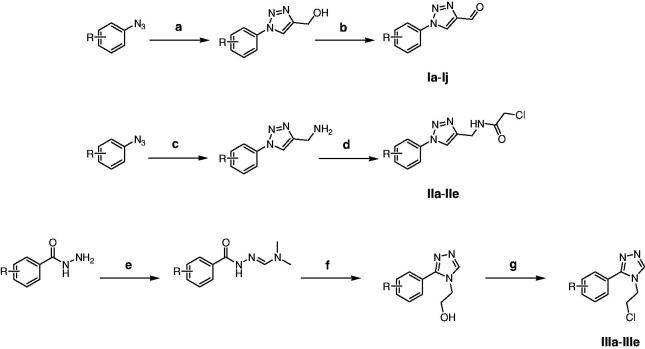
Reagents and conditions: (a) propargyl alcohol, CuSO_4_^.^5H_2_O, sodium ascorbate, t-BuOH/H_2_O (1:1), 30 °C. (b) MnO_2_, EtOAc, 70 °C. (c) propynylamine, CuSO_4_ 5H_2_O, sodium ascorbate, t-BuOH/H_2_O (1:1), 30 °C. (d) chloroacetyl chloride, Et_3_N, CH_2_Cl_2_, 30 °C. (e) DMFDMA, CH_3_CN, 60 °C. (f) 2-aminoethanol, CH_3_COOH, 90 °C. (g) sulfoxide chloride, CHCl_3_, 60 °C.

The compound **UA-1** was synthesised as per the protocol described in a previous study ^6^. **Ia-Ij** (different 1-phenyl-1*H*-1,2,3-triazole-4-carbaldehyde) and **IIa-IIe** (different 2-chloro-*N*-((1-phenyl-1*H*-1,2,3-triazol-4-yl)methyl)acetamide) were prepared as previously described^17^^,^^20^. **IIIa-IIIe** were prepared as per Scheme 1: different substitutions of benzoyl hydrazide (10 mmol) and N,N-Dimethylformamide dimethyl acetal (DMFDMA; 1.31 g, 11 mmol) were added to CH_3_CN (20 ml); the resulting mixture was stirred at 60 °C for 1 h. Then, 2-aminoethanol (1.22 g, 20 mmol) and CH_3_COOH (2.40 g, 40 mmol) were added, and the resulting mixture was stirred at 90 °C for 8–12 h. After confirming the reaction progress by TLC, the solvent was evaporated *in vacuo*. The mixture was then purified using silica gel column chromatography and eluted using a gradient of dichloromethane:methanol (100:1–40:1) to obtain different 2–(3-phenyl-4*H*-1,2,4-triazol-4-yl)ethanol derivatives. These products were placed in CHCl_3_ (20 ml) and 5 molar ratios of sulfoxide chloride was added. The mixture was stirred at 60 °C for 3 h. Upon completion, the solvent and excessive sulfoxide chloride was evaporated *in vacuo* to obtain different intermediates, which were used in the next step without further purification.

### 2.3. General procedure for synthesis of compound (**1a–1j**)

A mixture of **UA-1** (90.8 mg, 0.20 mmol), KOH (112 mg, 2.0 mmol) and different 1-phenyl-1*H*-1,2,3-triazole-4-carbaldehydes (0.21 mmol) was prepared in CH_3_CH_2_OH (10 ml) and stirred at 30 °C for 3–5 h. Progress of reaction was confirmed by TLC, following which the solvent was evaporated *in vacuo*. The mixture was neutralised with hydrochloric acid, extracted with 15 ml ethyl acetate, and then washed thrice with saline (5 ml). The final products were purified using preparative HPLC equipped with a C-18 column. A gradient elution was performed with tetrahydrofuran and water as the mobile phase and was monitored at 220 nm and 254 nm. ^1^H and ^13^C-NMR spectra of all the target compounds are available in the Supplementary materials.

#### (1S,2R,4aS,6aS,6bR,8aR,12aR,12bR,14bS,E)-1,2,6a,6b,9,9,12a-heptamethyl-10-oxo-11-((1-phenyl-1H-1,2,3-triazol-4-yl)methylene)-1,3,4,5,6,6a,6b,7,8,8a,9,10,11,12,12a,12b,13,14b-octadecahydropicene-4a(2H)-carboxylic acid (**1a**)

White solid; yield, 78%; m.p. >250 °C; ^1^H-NMR (CDCl_3_, 300 MHz, ppm): δ 8.09 (s, 1H, triazole-H), 7.80 − 7.77 (m, 2H, Ar-H), 7.61 − 7.47 (m, 4H, Ar–H, –CO–C = CH–), 5.35 (s, 1H, C_12_–H), 3.54 (d, *J* = 17.4 Hz, 1H, C_1_–He), 2.48 (d, *J* = 18.0 Hz, 1H, C_1_–Ha), 2.27 − 2.18 (m, 2H), 2.10 − 2.00 (m, 2H), 1.94 − 1.89 (m, 1H), 1.83 − 1.69 (m, 4H), 1.58 − 1.52 (m, 4H), 1.45 − 1.27 (m, 5H), 1.19 − 1.15 (m, 9H), 0.99 − 0.92 (m, 9H), 0.89 − 0.85 (m, 4H). ^13^C-NMR (CDCl_3_, 75 MHz, ppm): δ 207.63, 183.98, 145.17, 137.87, 136.69, 135.66, 129.89 (2C), 129.04, 125.82, 123.69, 122.94, 120.59 (2C), 53.04, 52.70, 48.11, 45.10 (2C), 44.94, 42.19, 39.38, 39.18, 38.86, 36.73, 36.04, 32.09, 30.65, 29.73, 28.03, 24.11, 23.70, 23.46, 22.61, 21.17, 20.37, 17.15, 16.75, 15.75. ESI-HRMS (*m/z*): calculated for C_39_H_52_N_3_O_3_^+^ [M + H]^+^: 610.4003, found: 610.4001.

#### (1S,2R,4aS,6aS,6bR,8aR,12aR,12bR,14bS,E)-11-((1–(2-fluorophenyl)-1H-1,2,3-triazol-4-yl)methylene)-1,2,6a,6b,9,9,12a-heptamethyl-10-oxo-1,3,4,5,6,6a,6b,7,8,8a,9,10,11,12,12a,12b,13,14b-octadecahydropicene-4a(2H)-carboxylic acid (**1b**)

White solid; yield, 82%; m.p. >250 °C; ^1^H-NMR (CDCl_3_, 300 MHz, ppm): δ 8.20 (s, 1H, triazole-H), 8.05 − 7.99 (m, 1H, Ar–H) 7.54 − 7.33 (m, 4H, Ar–H, –CO–C = CH–), 5.35 (s, 1H, C_12_–H), 3.50 (d, *J* = 17.7 Hz, 1H, C_1_–He), 2.47 (d, *J* = 18.0 Hz, 1H, C_1_–Ha), 2.27 − 2.18 (m, 2H), 2.10 − 2.01 (m, 2H), 1.94 − 1.89 (m, 1H), 1.83 − 1.69 (m, 4H), 1.58 − 1.52 (m, 5H), 1.46 − 1.31 (m, 4H), 1.27 − 1.15 (m, 10H), 0.99 − 0.92 (m, 10H), 0.90 − 0.87 (m, 2H). ^13^C-NMR (CDCl_3_, 75 MHz, ppm): δ 207.55, 183.78, 154.98, 151.66, 144.91, 137.86, 135.75, 130.36, 125.83, 125.42, 124.72, 123.68, 117.30, 117.04, 53.02, 52.71, 48.10, 45.14, 45.11, 44.99, 42.19, 39.38, 39.18, 38.85, 36.73, 36.04, 32.09, 30.65, 29.71, 28.02, 24.12, 23.65, 23.45, 22.58, 21.17, 20.36, 17.10, 16.75, 15.77. ESI-HRMS (*m/z*): calculated for C_39_H_51_FN_3_O_3_^+^ [M + H]^+^: 628.3909, found: 628.3907.

#### (1S,2R,4aS,6aS,6bR,8aR,12aR,12bR,14bS,E)-11-((1–(3-fluorophenyl)-1H-1,2,3-triazol-4-yl)methylene)-1,2,6a,6b,9,9,12a-heptamethyl-10-oxo-1,3,4,5,6,6a,6b,7,8,8a,9,10,11,12,12a,12b,13,14b-octadecahydropicene-4a(2H)-carboxylic acid (**1c**)

White solid; yield, 80%; m.p. >250 °C; ^1^H-NMR (CDCl_3_, 300 MHz, ppm): δ 8.08 (s, 1H, triazole-H), 7.60 − 7.50 (m, 4H, Ar–H, –CO–C = CH–), 7.23 − 7.18 (m, 1H, Ar–H), 5.34 (s, 1H, C_12_–H), 3.54 (d, *J* = 16.5 Hz, 1H, C_1_–He), 2.48 (d, *J* = 16.8 Hz, 1H, C_1_–Ha), 2.16 − 2.19 (m, 2H), 2.09 − 2.01 (m, 2H), 1.93 − 1.90 (m, 1H), 1.83 − 1.69 (m, 4H), 1.57 − 1.52 (m, 4H), 1.45 − 1.27 (m, 5H), 1.19 − 1.15 (m, 9H), 0.99 − 0.94 (m, 9H), 0.87 (s, 4H). ^13^C-NMR (CDCl_3_, 125 MHz, ppm): δ 207.65, 183.40, 163.15, 145.41, 137.91, 137.81, 136.14, 131.38, 125.82, 123.28, 122.76, 115.97, 115.89, 108.39, 53.07, 52.76, 48.10, 45.14, 45.09, 44.94, 42.22, 39.40, 39.17, 38.87, 36.73, 36.06, 32.09, 30.65, 29.73, 28.02, 24.14, 23.71, 23.47, 22.62, 21.16, 20.39, 17.14, 16.73, 15.77. ESI-HRMS (*m/z*): calculated for C_39_H_51_FN_3_O_3_^+^ [M + H]^+^: 628.3909, found: 628.3906.

#### (1S,2R,4aS,6aS,6bR,8aR,12aR,12bR,14bS,E)-11-((1–(4-fluorophenyl)-1H-1,2,3-triazol-4-yl)methylene)-1,2,6a,6b,9,9,12a-heptamethyl-10-oxo-1,3,4,5,6,6a,6b,7,8,8a,9,10,11,12,12a,12b,13,14b-octadecahydropicene-4a(2H)-carboxylic acid (**1d**)

White solid; yield, 82%; m.p. >250 °C; ^1^H-NMR (CDCl_3_, 300 MHz, ppm): δ 8.04 (s, 1H, triazole-H), 7.79 − 7.74 (m, 2H, Ar–H), 7.51 (s, 1H, –CO–C = CH–), 7.31 − 7.25 (m, 2H, Ar–H), 5.34 (s, 1H, C_12_–H), 3.53 (d, *J* = 16.8 Hz, 1H, C_1_–He), 2.47 (d, *J* = 18.6 Hz, 1H, C_1_–Ha), 2.26 − 2.18 (m, 2H), 2.10 − 2.01 (m, 2H), 1.94 − 1.89 (m, 1H), 1.83 − 1.69 (m, 4H), 1.58 − 1.50 (m, 4H), 1.46 − 1.28 (m, 5H), 1.19 − 1.15 (m, 9H), 1.05 − 0.92 (m, 9H), 0.87 (s, 4H). ^13^C-NMR (CDCl_3_, 75 MHz, ppm): δ 207.62, 183.80, 145.28, 137.91, 135.85, 132.94, 126.92, 125.78, 123.50, 123.04, 122.65, 122.54, 117.05, 116.74, 53.05, 52.71, 48.10, 45.11, 45.08, 44.92, 42.19, 39.38, 39.17, 38.86, 36.73, 36.04, 32.08, 30.64, 29.72, 28.03, 24.11, 23.70, 23.46, 22.61, 21.16, 20.36, 17.14, 16.75, 15.75. ESI-HRMS (*m/z*): calculated for C_39_H_51_FN_3_O_3_^+^ [M + H]^+^: 628.3909, found: 628.3907.

#### (1S,2R,4aS,6aS,6bR,8aR,12aR,12bR,14bS,E)-11-((1–(2-chlorophenyl)-1H-1,2,3-triazol-4-yl)methylene)-1,2,6a,6b,9,9,12a-heptamethyl-10-oxo-1,3,4,5,6,6a,6b,7,8,8a,9,10,11,12,12a,12b,13,14b-octadecahydropicene-4a(2H)-carboxylic acid (**1e**)

White solid; yield, 81%; m.p. >250 °C; ^1^H-NMR (CDCl_3_, 300 MHz, ppm): δ 8.12 (s, 1H, triazole-H), 7.70 − 7.62 (m, 2H, Ar–H), 7.56 (s, 1H, –CO–C = CH–), 7.53 − 7.49 (m, 2H, Ar–H), 5.34 (s, 1H, C_12_–H), 3.45 (d, *J* = 16.8 Hz, 1H, C_1_–He), 2.45 (d, *J* = 17.4 Hz, 1H, C_1_–Ha), 2.26 − 2.16 (m, 2H), 2.11 − 2.01 (m, 2H), 1.94 − 1.89 (m, 1H), 1.82 − 1.68 (m, 4H), 1.58 − 1.52 (m, 4H), 1.49 − 1.27 (m, 5H), 1.20 − 1.15 (m, 9H), 0.99 − 0.96 (m, 5H), 0.94 − 0.92 (m, 3H), 0.90 − 0.85 (m, 5H). ^13^C-NMR (CDCl_3_, 75 MHz, ppm): δ 207.56, 183.51, 144.29, 137.91, 135.65, 134.60, 130.93 (2C), 128.52, 128.07, 127.63, 126.74, 125.79, 123.87, 53.01, 52.73, 48.10, 45.18, 45.11, 45.08, 42.21, 39.38, 39.18, 38.85, 36.71, 36.03, 32.09, 30.65, 29.72, 28.02, 24.13, 23.69, 23.45, 22.55, 21.15, 20.36, 17.09, 16.75, 15.82. ESI-HRMS (*m/z*): calculated for C_39_H_51_ClN_3_O_3_^+^ [M + H]^+^: 644.3613, found: 644.3609.

#### (1S,2R,4aS,6aS,6bR,8aR,12aR,12bR,14bS,E)-11-((1–(3,4-dichlorophenyl)-1H-1,2,3-triazol-4-yl)methylene)-1,2,6a,6b,9,9,12a-heptamethyl-10-oxo-1,3,4,5,6,6a,6b,7,8,8a,9,10,11,12,12a,12b,13,14b-octadecahydropicene-4a(2H)-carboxylic acid (**1f**)

White solid; yield, 79%; m.p. >250 °C; ^1^H-NMR (CDCl_3_, 300 MHz, ppm): δ 8.06 (s, 1H, triazole-H), 7.95 (s, 1H, Ar–H), 7.67 (s, 2H, Ar–H), 7.48 (s, 1H, –CO–C = CH–), 5.34 (s, 1H, C_12_–H), 3.54 (d, *J* = 15.7 Hz, 1H, C_1_–He), 2.47 (d, *J* = 15.4 Hz, 1H, C_1_–Ha), 2.27 − 2.16 (m, 2H), 2.10 − 2.00 (m, 2H), 1.95 − 1.89 (m, 1H), 1.83 − 1.69 (m, 4H), 1.58 − 1.50 (m, 4H), 1.45 − 1.27 (m, 5H), 1.19–1.52 (m, 9H), 1.07 − 1.04 (m, 1H), 1.00 − 0.97 (m, 3H), 0.94 − 0.92 (m, 5H), 0.87–0.81 (m, 4H). ^13^C-NMR (CDCl_3_, 75 MHz, ppm): δ 207.58, 183.48, 145.60, 137.93, 136.39, 135.67, 134.19, 133.24, 131.62, 125.76, 123.02, 122.59, 122.35, 119.46, 53.05, 52.73, 48.10, 45.14, 45.09, 44.94, 42.21, 39.38, 39.18, 38.85, 36.72, 36.05, 32.08, 30.64, 29.70, 28.01, 24.12, 23.69, 23.45, 22.61, 21.15, 20.35, 17.12, 16.75, 15.75. ESI-HRMS (*m/z*): calculated for C_39_H_50_Cl_2_N_3_O_3_^+^ [M + H]^+^: 678.3224, found: 678.3225.

#### (1S,2R,4aS,6aS,6bR,8aR,12aR,12bR,14bS,E)-11-((1–(2-bromophenyl)-1H-1,2,3-triazol-4-yl)methylene)-1,2,6a,6b,9,9,12a-heptamethyl-10-oxo-1,3,4,5,6,6a,6b,7,8,8a,9,10,11,12,12a,12b,13,14b-octadecahydropicene-4a(2H)-carboxylic acid (**1g**)

White solid; yield, 76%; m.p. >250 °C; ^1^H-NMR (CDCl_3_, 300 MHz, ppm): δ 8.09 (s, 1H, triazole-H), 7.82 − 7.80 (m, 1H, Ar–H) 7.64 − 7.41 (m, 4H, Ar–H, –CO–C = CH–), 5.34 (s, 1H, C_12_–H), 3.46 (d, *J* = 17.1 Hz, 1H, C_1_–He), 2.45 (d, *J* = 18.0 Hz, 1H, C_1_–Ha), 2.27 − 2.18 (m, 2H), 2.10 − 2.01 (m, 2H), 1.94 − 1.89 (m, 1H), 1.82 − 1.66 (m, 4H), 1.58 − 1.47 (m, 6H), 1.42 − 1.32 (m, 3H), 1.21 − 1.16 (m, 9H), 1.08 − 0.97 (m, 6H), 0.94 − 0.92 (m, 3H), 0.88 − 0.82 (m, 4H). ^13^C-NMR (CDCl_3_, 75 MHz, ppm): δ 207.62, 183.67, 145.42, 137.90, 137.52, 136.15, 135.76, 130.97, 129.10, 125.79, 123.26, 122.74, 120.86, 118.52, 53.05, 52.72, 48.10, 45.13, 45.09, 44.94, 42.10, 39.39, 39.18, 38.85, 36.73, 36.05, 32.08, 30.64, 29.71, 28.02, 24.12, 23.70, 23.46, 22.61, 21.15, 20.36, 17.12, 16.75, 15.75. ESI-HRMS (*m/z*): calculated for C_39_H_51_BrN_3_O_3_^+^ [M + H]^+^: 688.3108, found: 688.3106.

#### (1S,2R,4aS,6aS,6bR,8aR,12aR,12bR,14bS,E)-11-((1–(2-iodophenyl)-1H-1,2,3-triazol-4-yl)methylene)-1,2,6a,6b,9,9,12a-heptamethyl-10-oxo-1,3,4,5,6,6a,6b,7,8,8a,9,10,11,12,12a,12b,13,14b-octadecahydropicene-4a(2H)-carboxylic acid (**1h**)

White solid; yield, 75%; m.p. >250 °C; ^1^H-NMR (CDCl_3_, 300 MHz, ppm): δ 8.07 − 8.01 (m, 2H, Ar-H, triazole-H), 7.59 − 7.49 (m, 3H, Ar–H, –CO–C = CH–), 7.32 − 7.29 (m, 1H), 5.34 (s, 1H, C_12_–H), 3.44 (d, *J* = 17.7 Hz, 1H, C_1_–He), 2.46 (d, *J* = 17.7 Hz, 1H, C_1_–Ha), 2.27 − 2.19 (m, 2H), 2.11 − 2.00 (m, 2H), 1.95 − 1.89 (m, 1H), 1.82 − 1.68 (m, 4H), 1.58 − 1.52 (m, 4H), 1.46 − 1.27 (m, 5H), 1.20 − 1.15 (m, 9H), 1.08 − 0.97 (m, 7H), 0.93 − 0.91 (m, 3H), 0.87 (s, 3H). ^13^C-NMR (CDCl_3_, 75 MHz, ppm): δ 207.61, 183.68, 144.22, 140.43, 139.71, 137.94, 135.66, 131.64, 129.41, 127.80, 126.64, 125.74, 124.03, 93.56, 52.96, 52.73, 48.11, 45.21, 45.12(2C), 42.21, 39.38, 39.16, 38.85, 36.71, 36.05, 32.08, 30.65, 29.73, 28.00, 24.11, 23.76, 23.46, 22.54, 21.15, 20.35, 17.14, 16.75, 15.87. ESI-HRMS (*m/z*): calculated for C_39_H_51_IN_3_O_3_^+^ [M + H]^+^: 736.2970, found: 736.2968.

#### (1S,2R,4aS,6aS,6bR,8aR,12aR,12bR,14bS,E)-11-((1–(2-methoxyphenyl)-1H-1,2,3-triazol-4-yl)methylene)-1,2,6a,6b,9,9,12a-heptamethyl-10-oxo-1,3,4,5,6,6a,6b,7,8,8a,9,10,11,12,12a,12b,13,14b-octadecahydropicene-4a(2H)-carboxylic acid (**1i**)

White solid; yield, 77%; m.p. >250 °C; ^1^H-NMR (CDCl_3_, 300 MHz, ppm): δ 8.31 (s, 1H, triazole-H), 7.90 (dd, *J* = 7.8, 1.5 Hz, 1H, Ar–H), 7.59 (s, 1H, –CO–C = CH–), 7.47 (td, *J* = 8.4, 1.5 Hz, 1H, Ar–H), 7.19 − 7.12 (m, 2H, Ar–H), 5.35 (s, 1H, C_12_–H), 3.93 (s, 3H, Ar–OCH_3_), 3.43 (d, *J* = 17.4 Hz, 1H, C_1_–He), 2.43 (d, *J* = 16.5 Hz, 1H, C_1_–Ha), 2.27 − 2.19 (m, 2H), 2.11 − 2.02 (m, 2H), 1.94 − 1.89 (m, 1H), 1.84 − 1.69 (m, 4H), 1.58 − 1.52 (m, 4H), 1.46 − 1.27 (m, 5H), 1.20 − 1.15 (m, 9H), 1.07 − 0.96 (m, 7H), 0.93 − 0.91 (m, 3H), 0.88 (s, 3H). ^13^C-NMR (CDCl_3_, 75 MHz, ppm): δ 207.51, 183.73, 150.77, 143.93, 138.04, 134.74, 130.18, 126.93, 125.92, 125.70, 125.05, 124.74, 121.41, 112.32, 55.92, 52.96, 52.76, 48.12, 45.27, 45.18, 45.06, 42.24, 39.37, 39.15, 38.84, 36.70, 35.94, 32.12, 30.66, 29.77, 28.01, 24.13, 23.71, 23.44, 22.54, 21.15, 20.34, 17.04, 16.73, 15.82. ESI-HRMS (*m/z*): calculated for C_40_H_54_N_3_O_4_^+^ [M + H]^+^: 640.4109, found: 640.4106.

#### (1S,2R,4aS,6aS,6bR,8aR,12aR,12bR,14bS,E)-1,2,6a,6b,9,9,12a-heptamethyl-10-oxo-11-((1–(3,4,5-trimethoxyphenyl)-1H-1,2,3-triazol-4-yl)methylene)-1,3,4,5,6,6a,6b,7,8,8a,9,10,11,12,12a,12b,13,14b-octadecahydropicene-4a(2H)-carboxylic acid (**1j**)

White solid; yield, 76%; m.p. >250 °C; ^1^H-NMR (CDCl_3_, 300 MHz, ppm): δ 8.04 (s, 1H, triazole-H), 7.50 (s, 1H, –CO–C = CH–), 6.99 (s, 2H, Ar–H), 5.33 (s, 1H, C_12_–H), 3.97 − 3.92 (m, 9H, Ar–OCH_3_), 3.55 (d, *J* = 17.7 Hz, 1H, C_1_–He), 2.48 (d, *J* = 17.4 Hz, 1H, C_1_–Ha), 2.26 − 2.19 (m, 2H), 2.10 − 2.01 (m, 2H), 1.94 − 1.89 (m, 1H), 1.83 − 1.69 (m, 4H), 1.58 − 1.52 (m, 4H), 1.45 − 1.27 (m, 5H), 1.19 − 1.15 (m, 9H), 1.06 − 0.91 (m, 10H), 0.87 (s, 3H). ^13^C-NMR (CDCl_3_, 75 MHz, ppm): δ 207.63, 183.75, 154.01(2C), 145.15, 138.56, 137.93, 135.76, 132.46, 125.75, 123.49, 123.21, 98.44(2C), 61.07, 56.45(2C), 53.00, 52.73, 48.11, 45.17, 45.10, 45.06, 42.22, 39.37, 39.17, 38.84, 36.71, 35.96, 32.10, 30.65, 29.74, 28.01, 24.12, 23.72, 23.44, 22.58, 21.15, 20.36, 17.05, 16.74, 15.78. ESI-HRMS (*m/z*): calculated for C_42_H_58_N_3_O_6_^+^ [M + H]^+^: 700.4320, found: 700.4318.

### 2.4. General procedure for synthesis of compound (2a–2e)

A mixture of **UA** (91.2 mg, 0.20 mmol), K_2_CO_3_ (41.5 mg, 0.30 mmol) and different phenyl 1,2,3-triazole chloroacetamides (0.21 mmol) in CH_3_CN (15 ml) was stirred at 60 °C for 2–3 h. After confirming the reaction progress by TLC, the solvent was evaporated *in vacuo*. The mixture was dissolved in 15 ml ethyl acetate, and then washed thrice with saline (5 ml). The final products were purified using preparative HPLC equipped with a C-18 column. A gradient elution was performed with tetrahydrofuran and water as the mobile phase and monitored at 220 nm and 254 nm.

#### (1S,2R,4aS,6aS,6bR,8aR,12aR,12bR,14bS)-2-oxo-2-((1-phenyl-1H-1,2,3-triazol-4-yl)methylamino)ethyl-10-hydroxy-1,2,6a,6b,9,9,12a-heptamethyl-1,2,3,4,4a,5,6,6a,6b,7,8,8a,9,10,11,12,12a,12b,13,14b-icosahydropicene-4a-carboxylate (**2a**)

White powder; yield, 80%; m.p. 170–172 °C; ^1^H-NMR (CDCl_3_, 300 MHz, ppm): δ 8.04 (s, 1H, triazole-H), 7.76 (d, *J* = 7.8 Hz, 2H, Ar–H), 7.57 − 7.44 (m, 3H, Ar–H), 6.88 (t, *J* = 6.0 Hz, 1H, –CO–NH–), 5.27 (s, 1H, C_12_–H), 4.75 − 4.68 (m, 3H, –CO–O–CH_2_–, –CO–NH–CHe), 4.40 (d, *J* = 15.6 Hz, 1H, –CO–NH–CHa), 3.22 − 3.17 (m, 1H, C_3_–OH), 2.24 (d, *J* = 11.4 Hz, 1H), 2.12 − 2.02 (m, 1H), 1.93 − 1.84 (m, 1H), 1.75 − 1.67 (m, 4H), 1.64 − 1.62 (m, 2H), 1.58 − 1.44 (m, 6H), 1.41 − 1.22 (m, 6H), 1.15 − 1.13 (m, 1H), 1.09 (s, 3H), 0.98 − 0.97 (m, 7H), 0.90 − 0.88 (m, 4H), 0.77 − 0.74 (m, 5H), 0.70 − 0.66 (m, 1H), 0.63 (s, 3H). ^13^C-NMR (CDCl_3_, 75 MHz, ppm): δ 176.02, 167.81, 144.81, 139.23, 136.82, 129.82(2C), 128.99, 125.45, 120.72, 120.38(2C), 78.94, 62.82, 55.11, 52.90, 48.42, 47.37, 42.15, 39.43, 39.14, 38.78, 38.70, 38.44, 36.85, 36.70, 34.52, 32.77, 30.51, 28.10, 27.84, 27.13, 24.42, 23.62, 23.08, 21.10, 18.20, 16.98, 16.89, 15.57, 15.26. ESI-HRMS (*m/z*): calculated for C_41_H_59_N_4_O_4_^+^ [M + H]^+^: 671.4531, found: 671.4528.

#### (1S,2R,4aS,6aS,6bR,8aR,12aR,12bR,14bS)-2-((1–(4-chlorophenyl)-1H-1,2,3-triazol-4-yl)methylamino)-2-oxoethyl-10-hydroxy-1,2,6a,6b,9,9,12a-heptamethyl-1,2,3,4,4a,5,6,6a,6b,7,8,8a,9,10,11,12,12a,12b,13,14b-icosahydropicene-4a-carboxylate (**2b**)

White powder; yield, 82%; m.p. 176–178 °C; ^1^H-NMR (CDCl_3_, 300 MHz, ppm): δ 8.02 (s, 1H, triazole-H), 7.72 (d, *J* = 8.7 Hz, 2H, Ar–H), 7.53 (d, *J* = 8.7 Hz, 2H, Ar–H), 6.88 (t, *J* = 6.0 Hz, 1H, –CO–NH–), 5.28 (s, 1H, C_12_–H), 4.75 − 4.66 (m, 3H, –CO–O–CH_2_–, –CO–NH–CHe), 4.39 (d, *J* = 15.9 Hz, 1H, –CO–NH–CHa), 3.23 − 3.18 (m, 1H, C_3_–OH), 2.24 (d, *J* = 11.1 Hz, 1H), 2.13 − 2.03 (m, 1H), 1.96 − 1.86 (m, 1H), 1.80 − 1.67 (m, 5H), 1.64 − 1.46 (m, 8H), 1.42 − 1.24 (m, 6H), 1.16 − 1.13 (m, 1H), 1.09 (s, 3H), 1.01 − 0.94 (m, 8H), 0.90 − 0.88 (m, 3H), 0.82 − 0.79 (m, 3H), 0.75 (s, 2H), 0.71 − 0.67 (m, 1H), 0.63 (s, 2H). ^13^C-NMR (CDCl_3_, 75 MHz, ppm): δ 176.04, 167.96, 143.16, 139.26, 135.24, 134.94, 130.04(2C), 125.43, 121.59(2C), 120.89, 78.95, 62.77, 55.11, 52.91, 48.42, 47.38, 42.17, 39.44, 39.14, 38.78, 38.70, 38.48, 36.88, 36.70, 34.42, 32.79, 30.51, 28.10, 27.85, 27.13, 24.42, 23.62, 23.13, 21.11, 18.22, 16.98, 16.92, 15.58, 15.29. ESI-HRMS (*m/z*): calculated for C_41_H_58_ClN_4_O_4_^+^ [M + H]^+^: 705.4141, found: 705.4146.

#### (1S,2R,4aS,6aS,6bR,8aR,12aR,12bR,14bS)-2-((1–(4-methoxyphenyl)-1H-1,2,3-triazol-4-yl)methylamino)-2-oxoethyl-10-hydroxy-1,2,6a,6b,9,9,12a-heptamethyl-1,2,3,4,4a,5,6,6a,6b,7,8,8a,9,10,11,12,12a,12b,13,14b-icosahydropicene-4a-carboxylate (**2c**)

White powder; yield, 80%; m.p. 180–181 °C; ^1^H-NMR (CDCl_3_, 300 MHz, ppm): δ 7.94 (s, 1H, triazole-H), 7.65 (d, *J* = 9.0 Hz, 2H, Ar–H), 7.04 (d, *J* = 9.0 Hz, 2H, Ar–H), 6.87 (t, *J* = 6.0 Hz, 1H, –CO–NH–), 5.27 (s, 1H, C_12_–H), 4.74 − 4.66 (m, 3H, –CO–O–CH_2_–, –CO–NH–CHe), 4.40 (d, *J* = 15.9 Hz, 1H, –CO–NH–CHa), 3.89 (s, 3H, ph–OCH_3_), 3.23 − 3.17 (m, 1H, C_3_–OH), 2.24 (d, *J* = 10.5 Hz, 1H), 2.09 − 2.03 (m, 1H), 1.93 − 1.85 (m, 1H), 1.75 − 1.68 (m, 4H), 1.60 − 1.45 (m, 9H), 1.42 − 1.27 (m, 6H), 1.15 − 1.13 (m, 1H), 1.09 (s, 3H), 1.00 − 0.98 (m, 7H), 0.90 − 0.88 (m, 3H), 0.80 (s, 3H), 0.75 (s, 3H), 0.71 − 0.67 (m, 1H), 0.63 (s, 2H). ^13^C-NMR (CDCl_3_, 75 MHz, ppm): δ 176.03, 167.82, 160.02, 144.53, 139.15, 130.21, 125.47, 122.02(2C), 120.88, 114.83(2C), 78.93, 62.79, 55.65, 55.12, 52.88, 48.40, 47.38, 42.14, 39.43, 39.13, 38.77, 38.70, 38.46, 36.87, 36.69, 34.49, 32.78, 30.51, 28.10, 27.85, 27.15, 24.41, 23.61, 23.11, 21.10, 18.20, 16.97, 16.90, 15.59, 15.29. ESI-HRMS (*m/z*): calculated for C_42_H_61_N_4_O_5_^+^ [M + H]^+^: 701.4636, found: 701.4640.

#### (1S,2R,4aS,6aS,6bR,8aR,12aR,12bR,14bS)-2-oxo-2-((1-p-tolyl-1H-1,2,3-triazol-4-yl)methylamino)ethyl-10-hydroxy-1,2,6a,6b,9,9,12a-heptamethyl-1,2,3,4,4a,5,6,6a,6b,7,8,8a,9,10,11,12,12a,12b,13,14b-icosahydropicene-4a-carboxylate (**2d**)

White powder; yield, 81%; m.p. 186–187 °C; ^1^H-NMR (CDCl_3_, 300 MHz, ppm): δ 7.99 (s, 1H, triazole-H), 7.62 (d, *J* = 8.4 Hz, 2H, Ar–H), 7.33 (d, *J* = 8.4 Hz, 2H, Ar–H), 6.88 (t, *J* = 5.7 Hz, 1H, –CO–NH–), 5.26 (s, 1H, C_12_–H), 4.73 − 4.66 (m, 3H, –CO–O–CH_2_–, –CO–NH–CHe), 4.40 (d, *J* = 15.9 Hz, 1H, –CO–NH–CHa), 3.22 − 3.18 (m, 1H, C_3_–OH), 2.43 (s, 3H, ph–CH_3_), 2.24 (d, *J* = 11.1 Hz, 1H), 2.12–2.02 (m, 1H), 1.93 − 1.83 (m, 1H), 1.76 − 1.66 (m, 5H), 1.63 − 1.45 (m, 7H), 1.39 − 1.21 (m, 6H), 1.13 − 1.12 (m, 1H), 1.08 (s, 3H), 1.03 − 0.97 (m, 7H), 0.89 − 0.87 (m, 4H), 0.78 (s, 3H), 0.74 (s, 3H), 0.70 − 0.66 (m, 1H), 0.62 (s, 2H). ^13^C-NMR (CDCl_3_, 75 MHz, ppm): δ 175.99, 167.73, 144.76, 139.19, 139.00, 134.62, 130.26(2C), 125.46, 120.50, 120.26(2C), 78.92, 62.81, 55.11, 52.89, 48.40, 47.36, 42.14, 39.42, 39.13, 38.77, 38.69, 38.44, 36.85, 36.69, 34.63, 32.76, 30.50, 28.09, 27.82, 27.14, 24.42, 23.61, 23.08, 21.09(2C), 18.19, 16.97, 16.88, 15.55, 15.26. ESI-HRMS (*m/z*): calculated for C_42_H_61_N_4_O_4_^+^ [M + H]^+^: 685.4687, found: 685.4685.

#### (1S,2R,4aS,6aS,6bR,8aR,12aR,12bR,14bS)-2-((1–(3,4-dichlorophenyl)-1H-1,2,3-triazol-4-yl)methylamino)-2-oxoethyl-10-hydroxy-1,2,6a,6b,9,9,12a-heptamethyl-1,2,3,4,4a,5,6,6a,6b,7,8,8a,9,10,11,12,12a,12b,13,14b-icosahydropicene-4a-carboxylate (**2e**)

White powder; yield, 86%; m.p. 203–204 °C; ^1^H-NMR (CDCl_3_, 300 MHz, ppm): δ 8.03 (s, 1H, triazole-H), 7.93 (s, 1H, Ar–H), 7.63 (s, 2H, Ar–H), 6.88 (t, *J* = 6.3 Hz, 1H, –CO–NH–), 5.28 (s, 1H, C_12_–H), 4.76 − 4.66 (m, 3H, –CO–O–CH_2_–, –CO–NH–CHe), 4.39 (d, *J* = 15.9 Hz, 1H, –CO–NH–CHa), 3.23 − 3.18 (m, 1H, C_3_–OH), 2.24 (d, *J* = 11.1 Hz, 1H), 2.13–2.03 (m, 1H), 1.95 − 1.87 (m, 1H), 1.80 − 1.66 (m, 4H), 1.63 − 1.60 (m, 4H), 1.53 − 1.47 (m, 4H), 1.42 − 1.23 (m, 7H), 1.14 − 1.10 (m, 4H), 1.03 − 0.98 (m, 6H), 1.08 (s, 3H), 0.90 − 0.85 (m, 5H), 0.80 − 0.76 (m, 5H), 0.72 − 0.68 (m, 1H), 0.63 (s, 2H), 0.70 − 0.66 (m, 1H), 0.62 (s, 2H). ^13^C-NMR (CDCl_3_, 75 MHz, ppm): δ 175.99, 167.91, 145.51, 139.39, 135.90, 134.12, 133.03, 131.50, 125.38, 122.17, 120.58, 119.23, 78.92, 62.79, 55.10, 52.94, 48.43, 47.37, 42.19, 39.44, 39.14, 38.81, 38.70, 38.49, 36.88, 36.72, 34.59, 32.78, 30.49, 28.09, 27.82, 27.15, 24.42, 23.62, 23.13, 21.09, 18.21, 16.98, 16.93, 15.56, 15.28. ESI-HRMS (*m/z*): calculated for C_41_H_57_Cl_2_N_4_O_4_^+^ [M + H]^+^: 739.3751, found: 739.3753.

### 2.5. General procedure for synthesis of compound (**3a–3e**)

A mixture of **UA** (91.2 mg, 0.20 mmol), K_2_CO_3_ (41.5 mg, 0.30 mmol) and different 4–(2-chloroethyl)-3-phenyl-4*H*-1,2,4-triazoles (0.21 mmol) in CH_3_CN (15 ml) was stirred at 60 °C for 4–6 h. After confirming the reaction progress by TLC, the solvent was evaporated *in vacuo*. The mixture was dissolved in 15 ml ethyl acetate, and then washed thrice with saline (5 ml). Final products were purified by preparative HPLC equipped with a C-18 column. A gradient elution was performed with tetrahydrofuran and water as the mobile phase and monitored at 220 nm and 254 nm.

#### 2 –(3-phenyl-4H-1,2,4-triazol-4-yl)ethyl(1S,2R,4aS,6aS,6bR,8aR,12aR,12bR,14bS)-10-hydroxy-1,2,6a,6b,9,9,12a-heptamethyl-1,3,4,5,6,6a,6b,7,8,8a,9,10,11,12,12a,12b,13,14b-octadecahydropicene-4a(2H)-carboxylate (**3a**)

White powder; yield, 77%; m.p. 243–244 °C; ^1^H-NMR (CDCl_3_, 300 MHz, ppm): δ 8.33 (s, 1H, triazole-H), 7.64 − 7.54 (m, 5H, Ar–H), 5.17 (s, 1H, C_12_–H), 4.31 − 4.24 (m, 4H, –O–CH_2_CH_2_–N–), 3.24 − 3.21 (m, 1H, C_3_–OH), 2.16 (d, *J* = 11.7 Hz, 1H), 2.08 − 1.86 (m, 4H), 1.65 − 1.61 (m, 6H), 1.53 − 1.44 (m, 5H), 1.40 − 1.27 (m, 5H), 1.07 − 1.05 (m, 4H), 1.00 − 0.97 (m, 7H), 0.91 (s, 3H), 0.85 (d, *J* = 6.3 Hz, 3H), 0.79 (s, 3H), 0.72 (d, *J* = 10.2 Hz, 1H), 0.63 (s, 3H). ^13^C-NMR (CDCl_3_, 75 MHz, ppm): δ 177.06, 153.94, 144.18, 137.82, 130.34, 129.07 (2C), 128.97 (2C), 126.56, 125.97, 78.96, 62.39, 55.16, 52.88, 48.28, 47.44, 44.00, 41.98, 39.48, 39.00, 38.85, 38.73, 38.55, 36.95, 36.68, 32.85, 30.46, 28.13, 27.89, 27.20, 24.22, 23.58, 23.23, 21.09, 18.27, 17.01, 16.96, 15.62, 15.41. ESI-HRMS (*m/z*): calculated for C_40_H_58_N_3_O_3_^+^ [M + H]^+^: 628.4473, found: 628.4470.

#### 2 –(3-(4-chlorophenyl)-4H-1,2,4-triazol-4-yl)ethyl(1S,2R,4aS,6aS,6bR,8aR,12aR,12bR,14bS)-10-hydroxy-1,2,6a,6b,9,9,12a-heptamethyl-1,3,4,5,6,6a,6b,7,8,8a,9,10,11,12,12a,12b,13,14b-octadecahydropicene-4a(2H)-carboxylate (**3b**)

White powder; yield, 79%; m.p. 245–246 °C; ^1^H-NMR (CDCl_3_, 300 MHz, ppm): δ 8.32 (s, 1H, triazole-H), 7.61 (d, *J* = 8.7 Hz, 2H, Ar–H), 7.53 (d, *J* = 8.7 Hz, 2H, Ar–H), 5.16 (s, 1H, C_12_–H), 4.29 − 4.25 (m, 4H, –O–CH_2_CH_2_–N–), 3.24 − 3.19 (m, 1H, C_3_–OH), 2.14 (d, *J* = 11.1 Hz, 1H), 2.04–1.95 (m, 1H), 1.93 − 1.81 (m, 2H), 1.69 − 1.66 (m, 3H), 1.63 − 1.55 (m, 6H), 1.51 − 1.43 (m, 4H), 1.36 − 1.27 (m, 5H), 1.07 − 1.04 (m, 4H), 1.00 − 0.96 (m, 6H), 0.91 (s, 3H), 0.86 − 0.84 (m, 3H), 0.79 (s, 3H), 0.71 (d, *J* = 11.7 Hz, 1H), 0.61 (s, 3H). ^13^C-NMR (CDCl_3_, 75 MHz, ppm): δ 177.00, 144.33, 144.29, 137.80, 137.07, 130.30(2C), 129.52(2C), 125.97, 124.43, 78.96, 62.12, 55.16, 52.89, 48.30, 47.42, 44.33, 41.99, 39.48, 38.99, 38.85, 38.73, 38.54, 36.94, 36.69, 32.84, 30.43, 28.13, 27.88, 27.19, 24.22, 23.57, 23.22, 21.08, 18.27, 17.02, 16.96, 15.62, 15.41. ESI-HRMS (*m/z*): calculated for C_40_H_57_ClN_3_O_3_^+^ [M + H]^+^: 662.4083, found: 662.4081.

#### 2 –(3-(4-fluorophenyl)-4H-1,2,4-triazol-4-yl)ethyl(1S,2R,4aS,6aS,6bR,8aR,12aR,12bR,14bS)-10-hydroxy-1,2,6a,6b,9,9,12a-heptamethyl-1,3,4,5,6,6a,6b,7,8,8a,9,10,11,12,12a,12b,13,14b-octadecahydropicene-4a(2H)-carboxylate (**3c**)

White powder; yield, 85%; m.p. 221–222 °C; ^1^H-NMR (CDCl_3_, 300 MHz, ppm): δ 8.32 (s, 1H, triazole-H), 7.68–7.63 (m, 2H, Ar–H), 7.27–7.22 (m, 2H, Ar–H), 5.17 (s, 1H, C_12_–H), 4.28–4.25 (m, 4H, –O–CH_2_CH_2_–N–), 3.25 − 3.21 (m, 1H, C_3_–OH), 2.16 (d, *J* = 11.1 Hz, 1H), 2.04 − 1.98 (m, 1H), 1.91 − 1.85 (m, 2H), 1.69 − 1.63 (m, 4H), 1.56 − 1.43 (m, 7H), 1.36 − 1.25 (m, 6H), 1.08 − 1.05 (m, 4H), 1.00 − 0.97 (m, 7H), 0.91 (s, 3H), 0.87 − 0.84 (m, 3H), 0.79 (s, 3H), 0.72 (d, *J* = 10.5 Hz, 1H), 0.62 (s, 3H). ^13^C-NMR (CDCl_3_, 75 MHz, ppm): δ 177.00, 160.28, 153.00, 144.16, 137.81, 131.25, 130.14, 125.97, 122.05, 116.62, 116.32, 78.94, 62.14, 55.16, 52.88, 48.30, 47.42, 44.18, 41.99, 39.48, 38.99, 38.85, 38.73, 38.54, 36.94, 36.70, 32.85, 30.43, 28.13, 27.87, 27.19, 24.22, 23.57, 23.22, 21.07, 18.26, 17.01, 16.96, 15.62, 15.40. ESI-HRMS (*m/z*): calculated for C_40_H_57_FN_3_O_3_^+^ [M + H]^+^: 646.4378, found: 646.4373.

#### 2 –(3-(4-nitrophenyl)-4H-1,2,4-triazol-4-yl)ethyl(1S,2R,4aS,6aS,6bR,8aR,12aR,12bR,14bS)-10-hydroxy-1,2,6a,6b,9,9,12a-heptamethyl-1,3,4,5,6,6a,6b,7,8,8a,9,10,11,12,12a,12b,13,14b-octadecahydropicene-4a(2H)-carboxylate (**3d**)

White powder; yield, 82%; m.p. >250 °C; ^1^H-NMR (CDCl_3_, 300 MHz, ppm): δ 8.43 − 8.39 (m, 3H, triazole-H, Ar–H), 7.93 (d, *J* = 8.7 Hz, 2H, Ar–H), 5.15 (s, 1H, C_12_–H), 4.33 (dd, *J* = 17.7, 5.1 Hz, 4H, –O–CH_2_CH_2_–N–), 3.24 − 3.19 (m, 1H, C_3_–OH), 2.13 (d, *J* = 11.1 Hz, 1H), 2.07 − 1.97 (m, 1H), 1.93 − 1.77 (m, 2H), 1.63 − 1.59 (m, 5H), 1.56 − 1.45 (m, 6H), 1.35 − 1.25 (m, 6H), 1.07 (s, 4H), 1.00 − 0.96 (m, 7H), 0.90 (s, 3H), 0.86 − 0.84 (m, 3H), 0.79 (s, 3H), 0.71 (d, *J* = 11.1 Hz, 1H), 0.60 (s, 3H). ^13^C-NMR (CDCl_3_, 75 MHz, ppm): δ 176.97, 151.99, 148.83, 144.91, 137.76, 132.67, 129.82(2C), 126.00, 124.28(2C), 78.92, 62.08, 55.13, 52.90, 48.33, 47.39, 44.28, 41.98, 39.47, 38.97, 38.87, 38.72, 38.52, 36.92, 36.72, 32.82, 30.40, 28.12, 27.86, 27.16, 24.24, 23.56, 23.19, 21.06, 18.24, 17.02, 16.96, 15.62, 15.39. ESI-HRMS (*m/z*): calculated for C_40_H_57_N_4_O_5_^+^ [M + H]^+^: 673.4323, found: 673.4320.

#### 2 –(3-(4-methoxyphenyl)-4H-1,2,4-triazol-4-yl)ethyl(1S,2R,4aS,6aS,6bR,8aR,12aR,12bR,14bS)-10-hydroxy-1,2,6a,6b,9,9,12a-heptamethyl-1,3,4,5,6,6a,6b,7,8,8a,9,10,11,12,12a,12b,13,14b-octadecahydropicene-4a(2H)-carboxylate (**3e**)

White powder; yield, 76%; m.p. 233 − 235 °C; ^1^H-NMR (CDCl_3_, 300 MHz, ppm): δ 8.29 (s, 1H, triazole-H), 7.57 (d, *J* = 9.0 Hz, 2H, Ar–H), 7.04 (d, *J* = 8.7 Hz, 2H, Ar–H), 5.17 (s, 1H, C_12_–H), 4.28 − 4.24 (m, 4H, –O–CH_2_CH_2_–N–), 3.89 (s, 3H, ph-OCH_3_), 3.24 − 3.19 (m, 1H, C_3_–OH), 2.16 (d, *J* = 11.1 Hz, 1H), 2.03 − 1.97 (m, 1H), 1.91 − 1.84 (m, 2H), 1.66 − 1.56 (m, 7H), 1.52 − 1.43 (m, 5H), 1.40 − 1.25 (m, 6H), 1.07 (s, 4H), 1.00 − 0.96 (m, 6H), 0.91 (s, 3H), 0.86 − 0.84 (m, 3H), 0.79 (s, 3H), 0.72 (d, *J* = 11.4 Hz, 1H), 0.62 (s, 3H). ^13^C-NMR (CDCl_3_, 75 MHz, ppm): δ 176.98, 161.89, 152.04, 143.85, 137.84, 133.86, 130.79(2C), 125.93, 114.81(2C), 78.97, 61.95, 55.51, 55.15, 52.85, 48.29, 47.42, 44.17, 41.98, 39.46, 38.99, 38.81, 38.73, 38.54, 36.94, 36.67, 32.84, 30.43, 28.13, 27.87, 27.20, 24.21, 23.56, 23.21, 21.08, 18.26, 17.02, 16.95, 15.63, 15.41. ESI-HRMS (*m/z*): calculated for C_41_H_60_N_3_O_4_^+^ [M + H]^+^: 658.4578, found: 658.4580.

### 2.6. In vitro anti-T. gondii activity

The cytotoxicity of compounds was determined using the previously published thiazolyl blue-based colorimetric method. For this, HeLa cells were used as host cells and their ability to resist invasion by *T. gondii* RH strain tachyzoites *in vitro* was checked. The cells were plated in 96-well plates at an appropriate density to ensure exponential growth throughout the experimental period (3 × 10^3^ cells per well) and then allowed to adhere for 24 h at 37 °C. The cells were infected with *T. gondii* (1.5 × 10^4^ tachyzoites/well), followed by incubation for 24 h. All compounds were prepared in dimethyl sulfoxide (DMSO) at a stock concentration of 100 mM. Serial dilutions (1–1000 μM) of each compound were tested. Spiramycin was used as a positive control. After 24 h of incubation, 10 μL of MTT solution were added to each well and cells were incubated for a further 2 h. The optical density (OD) was read on a microplate reader at a wavelength of 492 nm. The IC_50_ in HeLa cells, IC_50_ in *T. gondii* and selectivity index were calculated using Microsoft Excel.

### 2.7. In vivo anti-T. gondii activity

Thirty female KM mice were used to establish an animal model of acute *T. gondii* infection. These were randomly divided into five groups: infected untreated, normal, infected with spiramycin treatment, infected with **1e** treatment and infected with **3d** treatment. Each group consisted of six mice. Four hours after infection, 100 mg/kg of the compounds was administered to the mice by gavage, once a day for 4 consecutive days, whereas the untreated group was administered the same dose of physiological saline. On the fifth day, blood from the eyes of mice was collected and they were sacrificed by cervical dislocation. Their abdominal cavity was rinsed with sterile physiological saline to collect the parasites/tachyzoites. These were counted under the light microscope, and the inhibition rate of parasites was calculated. The liver and spleen were dissected and liver and spleen indexes, serum alanine aminotransferase (ALT), aspartate aminotransferase (AST), and liver homogenate glutathione (GSH) and malonaldehyde (MDA) were determined.

### 2.8. Molecular docking

Molecular docking was performed using the Discovery Studio (DS) 2017 software. The protein and ligand samples were prepared, water molecules were deleted, and a DS Server added hydrogen. The docking result was treated with DS Client. In this study, three crystal structures of the proteins were selected for docking, PDB ID: 6BFA (calcium-dependent protein kinase 1)^21^, 1LII (adenosine kinase)^22^ and 3MB8 (purine nucleoside phosphorylase)^23^. The different xyz coordinates and radii of these proteins were defined as the binding site spheres. The output poses of the ligands generated were analysed using the LibDockScore function to find out the best complimentary match between the ligand and the receptor. The protocol, CDOCKER was used to perform the docking.

## 3. Results and discussion

### 3.1. Chemistry

**Scheme 2.**
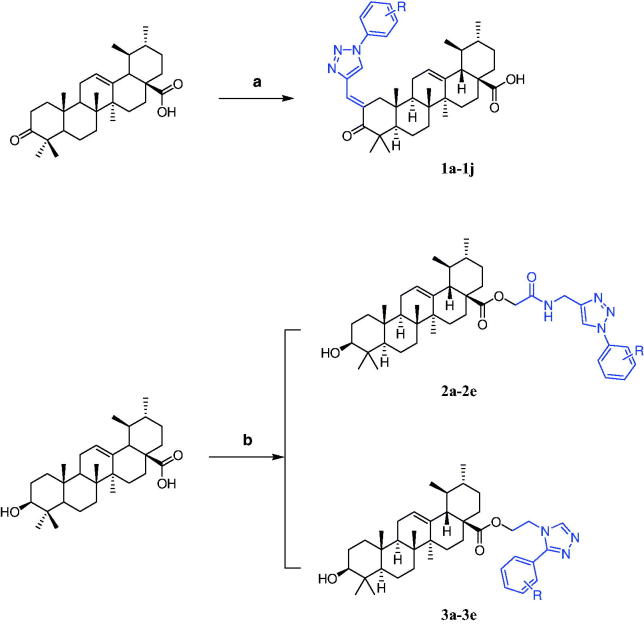
Reagents and conditions: (a) **Ia**–**Ij**, KOH, CH_3_CH_2_OH, 30 °C. (b) **IIa**–**IIe**, **IIIa**–**IIIe**, K_2_CO_3_, CH_3_CN, 60 °C.

Scheme 2 shows the procedure adopted to obtain target compounds. **UA-1** was obtained by Jones oxidation of **UA** at 0 °C. Compounds **1a**–**1j** were prepared by Claisen Schmidt condensation of **UA-1** with different aldehydes in the presence of ethanolic KOH at 30 °C. Good yields (76–82%) were obtained with this method. All other **UA** derivatives (**2a**–**2e** and **3a**–**3e**) were synthesised from various chlorinated derivatives via nucleophilic substitution in good to excellent yields (76–86%). Before biological evaluation, all target compounds were characterised via HRMS, ^1^H-NMR and ^13^C-NMR.

### 3.2. Evaluation of anti-T. gondii activity in vitro and preliminary structure-activity relationship

Selectivity index is a measure of specific resistance to *T. gondii*. As shown in Table 1, the SI value of the lead compound **UA** (0.62) was lower than that of the positive control drug spiramycin (0.72), indicating a certain degree of anti-*T. gondii* activity of **UA**. Among **UA** derivatives, nine compounds exhibited higher anti-*T. gondii* activity than **UA** alone (**1e**, **1f**, **1g**, **1h**, **1j**, **3a**, **3c**, **3d** and **3e**), and eight compounds exhibited an activity higher than spiramycin (**1e**, **1f**, **1g**, **1h**, **1j**, **3a**, **3c** and **3d**). Besides, with the exception of compound **3b**, the IC_50_ value of all other compounds was higher than that of **UA**, indicating these compounds to be less cytotoxic than **UA**. Similarly, compounds **1e**, **1f**, **1g**, **1h**, **1j** and **3d** displayed a higher anti-*T. gondii* activity and less cytotoxicity when compared with spiramycin.

**Table 1.** *In vitro T. gondii* growth inhibition and cytotoxicity on HeLa cells.

Compounds	R	IC_50_^a^ in HeLa cells (μM)	IC_50_^b^ in *T. gondii* (μM)	SI^c^
**1a**	-H	>1000	>1000	–
**1b**	2-F	419.6	711.9	0.59
**1c**	3-F	>1000	>1000	–
**1d**	4-F	>1000	>1000	–
**1e**	2-Cl	466.1	239.6	1.95
**1f**	3,4-Cl	408.0	462.1	0.88
**1g**	2-Br	240.4	301.7	0.80
**1h**	2-I	230.4	273.8	0.84
**1i**	2-OCH_3_	>1000	>1000	–
**1j**	3,4,5-OCH_3_	353.7	301.3	1.17
**2a**	-H	836.8	>1000	–
**2b**	4-Cl	328.7	>1000	–
**2c**	4-OCH_3_	>1000	>1000	–
**2d**	4-CH_3_	>1000	>1000	–
**2e**	3,4-Cl	>1000	>1000	–
**3a**	-H	101.5	88.0	1.15
**3b**	4-Cl	2.4	6.7	0.36
**3c**	4-F	88.2	61.4	1.44
**3d**	4-NO_2_	226.7	128.0	1.77
**3e**	4-OCH_3_	78.2	116.5	0.67
Spiramycin	–	189.0	262.2	0.72
Ursolic Acid	–	44.8	72.2	0.62

a IC_50_ in HeLa cells: Median toxicity dose, a measure of cytotoxicity against host cells.

b IC_50_ in *T. gondii*: Median inhibitory concentration, a measure of tachyzoite inhibition.

c SI: Selectivity index, a measure of efficacy, calculated by IC_50_ in HeLa cells/IC_50_ in *T. gondii*.

Compounds **1a**–**1j** are products of a reaction between **UA-1** and **Ia–Ij**. The anti-*T. gondii* activity of these compounds with different substitutions on the benzene ring was found to be in the following order: 2-Cl > 3,4,5-OCH_3_ > 3,4-Cl > 2-I > 2-Br > 2-F > 2-OCH_3_=H. Based on an overall comparison, we hypothesised that introduction of halogen substituents at the *ortho* position and electron-donating group at the 3,4,5-position of benzene ring could improve the anti-*T. gondii* activity. Compounds **2a**–**2e** were generated from **UA** and **IIa**–**IIe**. Unfortunately, all of these compounds lost their anti-*T. gondii* activity. Among the compounds **3a**–**3e,** which react with **IIIa**–**IIIe**, four compounds showed considerably higher anti-*T. gondii* activity. It seems that the anti-*T. gondii* ability was enhanced after the introduction of strong electron-withdrawing group (–F, –NO_2_) to the *para* position of the benzene ring. Based on these findings, we decided to conduct an in-depth study of anti-*T. gondii* activity of compounds **1e** and **3d** in mice, owing to their strong anti-*T. gondii* activity *in vitro*.

### 3.3. Number of tachyzoites in vivo

**Figure 2.**
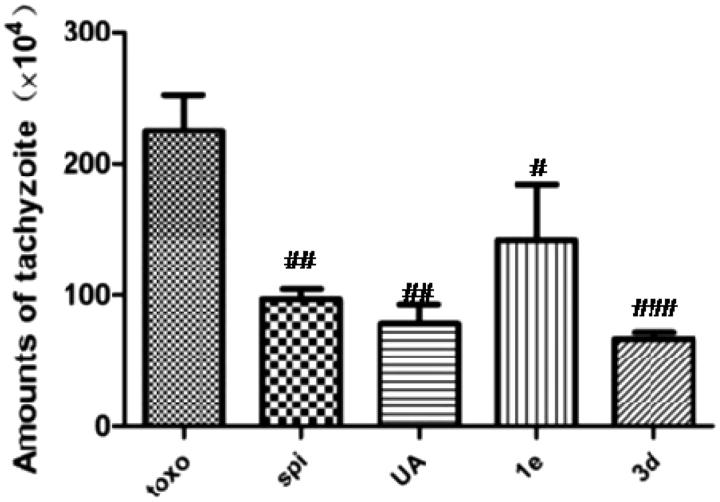
Effect of compounds on the number of tachyzoites in KM mice, *n* = 6, #*p* < .05, ##*p* < .01, ###*p* < .001 compared with toxo group.

As shown in Table 2 and Figure 2, the number of intraperitoneal tachyzoites in untreated KM mice was 225 × 10^4^. After treatment with 100 mg/kg of different compounds, this number decreased to varying degrees in the ascitic fluid of spiramycin-, **UA**-, compound **1e**- and compound **3d**-treated mice, with inhibitory rates being 56.8%, 65.4%, 37.0% and 70.4%, respectively. It is clearly interpreted from these data that treatment with compound **3d** could significantly decrease the number of tachyzoites in *T. gondii*-infected KM mice (*p* < .001). It even showed better anti-*T. gondii* activity than spiramycin and **UA***in vivo*.

**Table 2.** *In vivo* anti-*T. gondii* activity.

Groups	Amount of tachyzoite (×10^4^)
Toxo^a^	225.0
Spi^b^	97.2
**UA**	77.9
**1e**	141.8
**3d**	66.6

a *T. gondii*-infected KM mice with no treatment.

b Spiramycin.

### 3.4. Liver and spleen indexes

**Figure 3.**
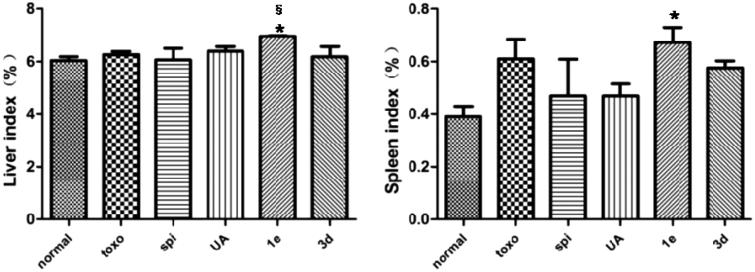
Effect of compounds on liver and spleen weights in *T. gondii*-infected KM mice, *p < .05 compared with normal group; §*p* < .05 compared with spi group.

Liver and spleen indexes were used to evaluate the protective effect of drugs on viscera. As shown in Figure 3, compared with the normal group, the liver index of the mice infected with *T. gondii* increased only slightly. Although a significant increase in the spleen index was observed in mice infected with *T. gondii*, this increase was subjugated by treatment with spiramycin, **UA** or compound **3d**. However, changes in spleen index did not show any statistically significant differences.

### 3.5. ALT and AST

**Figure 4.**
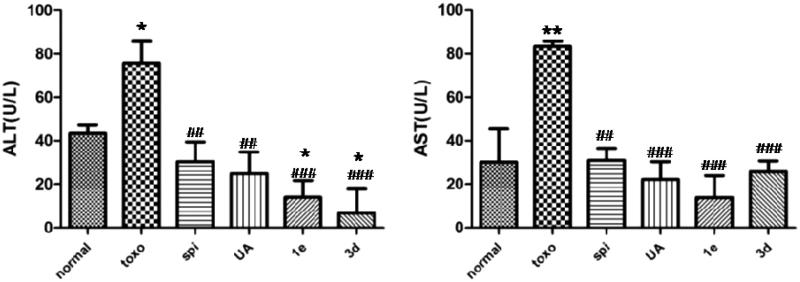
Effect of compounds on ALT and AST levels in *T. gondii*-infected KM mice, *p < .05, **p < .01 compared with normal group; ##*p* < .01, ###*p* < .001 compared with toxo group.

Levels of serum ALT and AST act as indicators of hepatotoxicity. To further study the toxicity of these compounds, ALT and AST levels in the serum of KM mice after infection with *T. gondii* were measured (Figure 4). *T. gondii* infection resulted in a significant elevation of serum ALT and AST levels as compared with the normal group. Treatment with **UA**, **1e** and **3d** led to a striking reduction in these levels as compared with the untreated group. These results indicated **UA, 1e** and **3d** could provide resistance against *T. gondii*-mediated hepatotoxicity.

### 3.6. MDA and GSH

**Figure 5.**
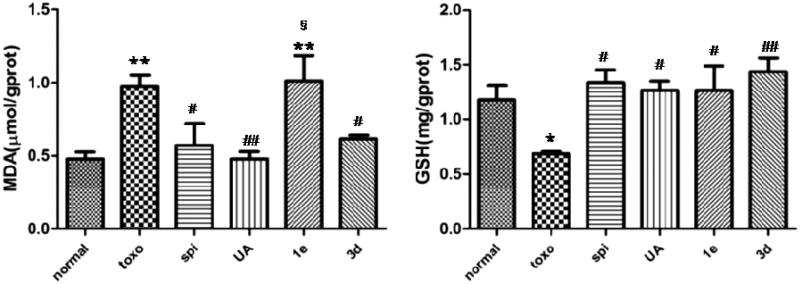
Effect of compounds on MDA and GSH levels in *T. gondii*-infected KM mice, *p < .05, **p < .01 compared with normal group; #*p* < .05, ##*p* < .01 compared with toxo group; §*p* < .05 compared with spi group.

Free radicals generated within cells cause peroxidation of lipids, resulting in the formation of MDA, which, in turn, causes cross-linking and polymerisation of proteins, nucleic acids and other macromolecules, thereby exerting cytotoxicity. As can be seen from the data in Figure 5, the untreated group had a higher MDA content compared with the normal group (*p* < .01), whereas levels of MDA significantly decreased after treatment with spiramycin, **UA** or compound **3d**. GSH is an important antioxidant that scavenges the free radicals in the body. It combines with free radicals and heavy metals, thereby converting them to harmless substances that are excreted from the body^24^. Compared with the normal group, the GSH content in the untreated group was significantly decreased (*p* < .05). However, compounds **3d** and **1e** could significantly increase the GSH content as compared to the untreated group, and had a similar efficacy to **UA** and spiramycin. These results implied that the anti-oxidative effects of **UA** and compound **3d** were comparable to that of spiramycin.

### 3.7. Molecular docking analysis

**Figure 6.**
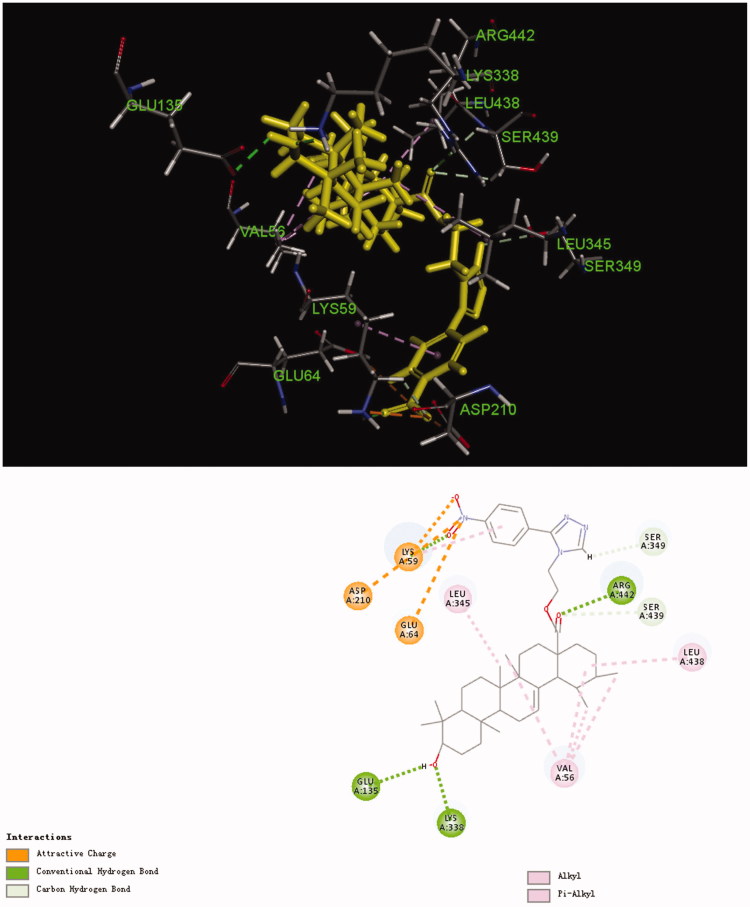
Computer modelling of compound **3d** binding to calcium-dependent protein kinase 1 (6BFA). Compound **3d** was coloured in yellow.

TgCDPK1 plays a crucial role in the motility and gliding of *T. gondii*, as well as the adenosine kinase and purine nucleoside phosphorylase are key purine metabolic enzymes from the *T. gondii*^21–23^. Our *in vivo* study revealed compound **3d** to significantly inhibit the proliferation of tachyzoites in the abdominal cavity of KM mice. Therefore, these three enzymes related to *T. gondii* metabolism were selected for molecular docking study and to determine possible targets with their specific modes of action (Table 3). Interestingly, only TgCDPK1 (6BFA) could be docked and expressed a high binding energy for the ligand. The CDOCKER interaction energy of compound **3d** was 58.0486, slightly higher than that of **UA**, which was consistent with the result of *in vivo*. Figure 6 illustrates the binding mode of compound **3d** in its active site; it was held in the active pocket of TgCDPK1 through a combination of interactions with TgCDPK1. The nitro group of compound **3d** interacted with the –NH_3_^+^ group of Lys-A59, –COOH group of Asp-A210 and –COOH group of Glu-A64 via three important attractive charges. These interactions may explain the strong anti-*T. gondii* activity exhibited by compound **3d** in this series. Meanwhile, the carbonyl group of **UA** interacted with the = NH moiety of Arg-A442 and the –CH_2_– moiety of Ser-A439 via hydrogen and carbon–hydrogen bonds, respectively, whereas the –OH group of **UA** interacted with the –COOH group of Glu-A135 and the –NH_2_ group of Lys-A338 via two hydrogen bonds. In addition, the 1,2,4-triazole moiety formed one carbon–hydrogen bond with Ser-A439 residue. We also observed that **UA** entered into an alkyl interaction with amino acid residues Val-A56, Leu-A345 and Leu-A438.

**Table 3.** Scores of **UA** and compound **3d** docked to different enzymes.

Enzyme (PDB ID)	CDOCKER interaction energy	
	**UA**	Compound **3d**
6BFA	54.6825	58.0486
1LII	No docking	No docking
3MB8	No docking	No docking

**Figure 7.**
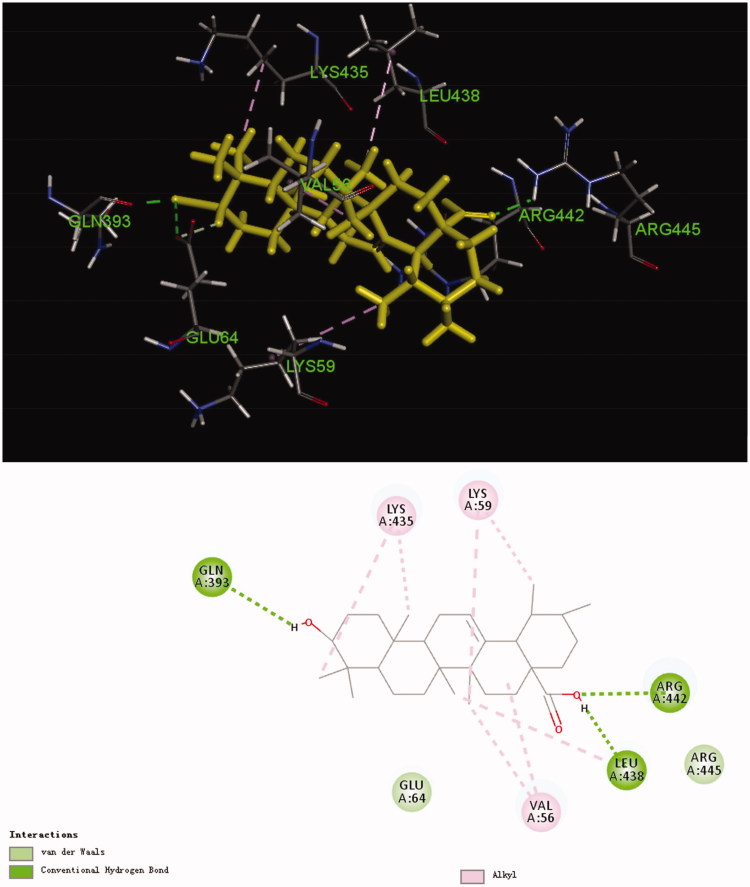
Computer modelling of **UA** binding to calcium-dependent protein kinase 1 (6BFA). **UA** was coloured in yellow.

In order to better reflect the advantage of compound **3d**, we also performed the molecular docking analysis of **UA**. As shown in Figure 7, three similar conventional hydrogen bonds are observed with residue Gln-A393, Leu-A438 and Arg-A442. However, compared with compound **3d**, some significant chemical bonds such as attractive charges are missing. This may explain why compound **3d** has better anti-*T. gondii* activity than **UA**. These results indicate compound **3d** to possess a strong binding affinity for the enzyme and therefore could act as a possible TgCDPK1 inhibitor.

## 4. Conclusions

In the present study, 20 novel **UA** derivatives were synthesised and examined for their anti-*T. gondii* properties. Most of these compounds displayed some anti-*T. gondii* activity, with a less cytotoxicity than **UA***in vitro*. The compound **3d** exhibited the most potent anti-*T. gondii* activity *in vivo* and was superior to **UA** and spiramycin. Docking study confirmed the anti-*T. gondii* activity of **3d**, as evident by the presence of three significant attractive charges and three hydrogen bonds in it that play a crucial role in its binding to the active site of TgCDPK1. Based on these findings, we conclude that compound **3d** may serve as a potential candidate for developing effective and anti-*T. gondii* drugs with fewer side-effects.

## Supplementary Material

Supplemental Material
