# Supplementary material for: Synthesis and biological evaluation of ursolic acid derivatives bearing triazole moieties as potential anti-Toxoplasma gondii agents
Source: J Enzyme Inhib Med Chem. 2019 Mar 5;34(1):761–72. doi: 10.1080/14756366.2019.1584622 (PMC6407578; doi:10.1080/14756366.2019.1584622)
Supplement: Supplemental Material [file IENZ_A_1584622_SM1592.pdf]

Supplementary File

Synthesis and biological evaluation of ursolic acid derivatives bearing triazole moieties as potential anti-*Toxoplasma gondii* agents

Tian Luan[a#], Chunmei Jin[a#], Chun-Mei Jin[a]*, Guo-Hua Gong[b,c]* & Zhe-Shan Quan[a]*

[a]*Key Laboratory of Natural Resources and Functional Molecules of the Changbai Mountain, Affiliated Ministry of Education, College of Pharmacy, Yanbian University, Yanji, Jilin, 133002, China*
[b]*First Clinical Medical College of Inner Mongolia University for Nationalities, Tongliao, P.R. China*
[c]*Inner Mongolia Key Laboratory of Mongolian Medicine Pharmacology for Cardio-Cerebral Vascular System, Inner Mongolia University for Nationalities, Tongliao, P.R. China*

***Corresponding author: Tel: + 86 433 243-6020; Fax: + 86 433 243-6020.**

E-mail: zsquan@ybu.edu.cn (Z. S. Quan).

***Corresponding author: E-mail: gongguohua0211@163.com (G. H. Gong).**

***Corresponding author: E-mail: cmjin@ybu.edu.cn (C. M. Jin).**

[#]These authors contributed equally to this work.

Table of contents:

^{1}H NMR and ^{13}C NMR spectra of compound **1a** page 3
^{1}H NMR and ^{13}C NMR spectra of compound **1b** page 4
^{1}H NMR and ^{13}C NMR spectra of compound **1c** page 5
^{1}H NMR and ^{13}C NMR spectra of compound **1d** page 6
^{1}H NMR and ^{13}C NMR spectra of compound **1e** page 7
^{1}H NMR and ^{13}C NMR spectra of compound **1f** page 8
^{1}H NMR and ^{13}C NMR spectra of compound **1g** page 9
^{1}H NMR and ^{13}C NMR spectra of compound **1h** page 10
^{1}H NMR and ^{13}C NMR spectra of compound **1i** page 11

^{1}H NMR and ^{13}C NMR spectra of compound **1j** page 12
^{1}H NMR and ^{13}C NMR spectra of compound **2a** page 13
^{1}H NMR and ^{13}C NMR spectra of compound **2b** page 14
^{1}H NMR and ^{13}C NMR spectra of compound **2c** page 15
^{1}H NMR and ^{13}C NMR spectra of compound **2d** page 16
^{1}H NMR and ^{13}C NMR spectra of compound **2e** page 17
^{1}H NMR and ^{13}C NMR spectra of compound **3a** page 18
^{1}H NMR and ^{13}C NMR spectra of compound **3b** page 19
^{1}H NMR and ^{13}C NMR spectra of compound **3c** page 20
^{1}H NMR and ^{13}C NMR spectra of compound **3d** page 21
^{1}H NMR and ^{13}C NMR spectra of compound **3e** page 22
^{1}H-NMR spectra of intermediate **IIId** page 23

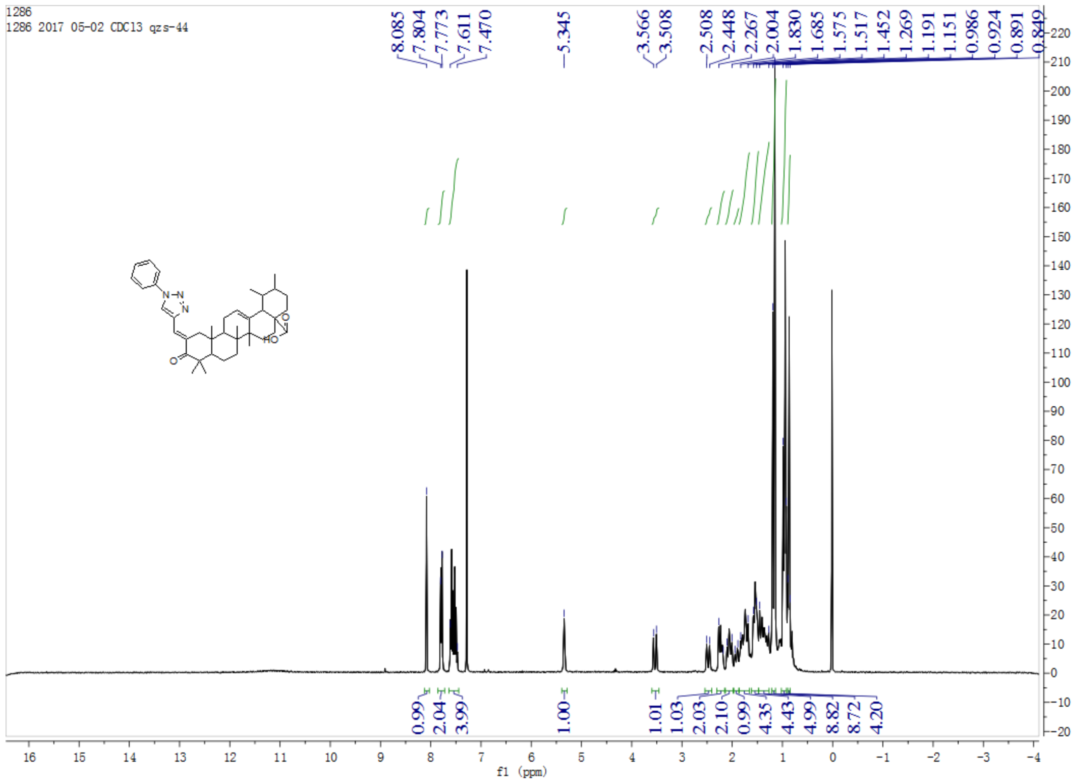

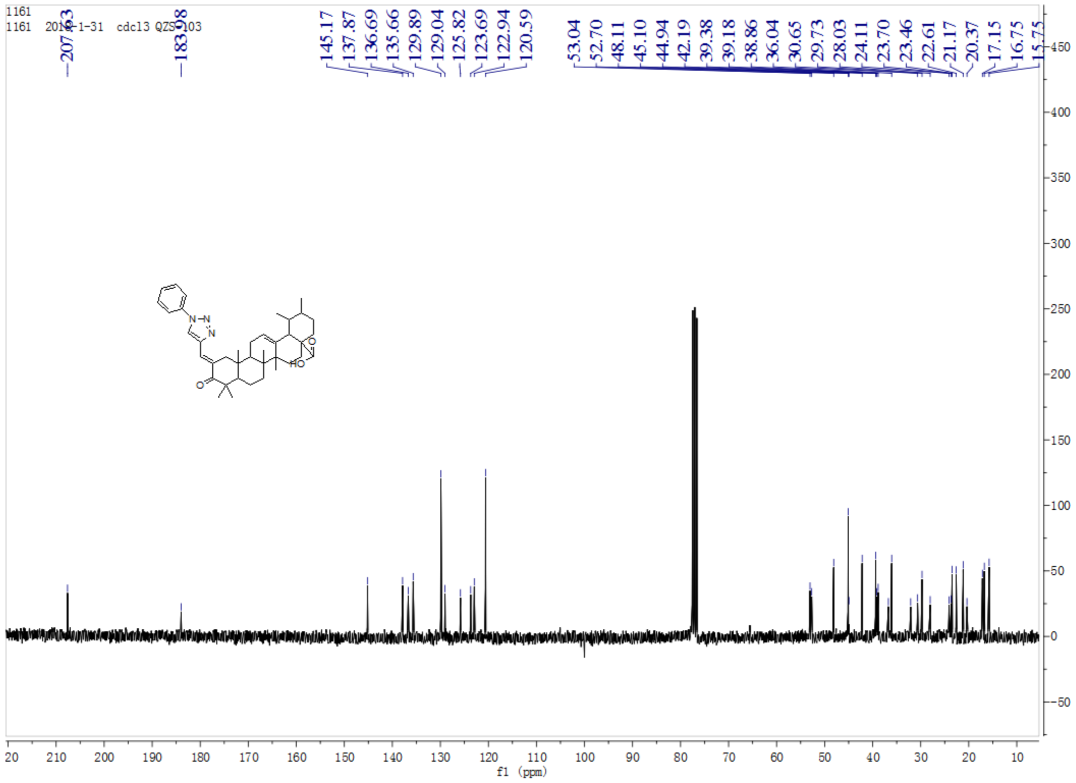

^{1}H NMR and ^{13}C NMR spectra of compound **1a**

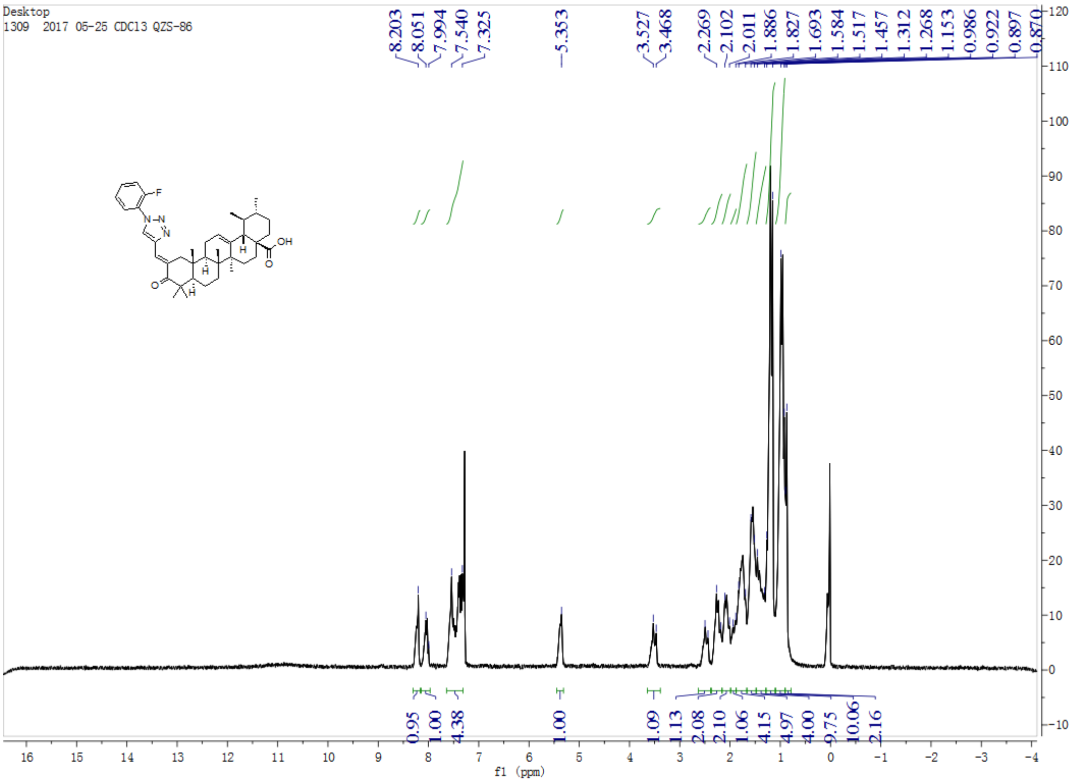

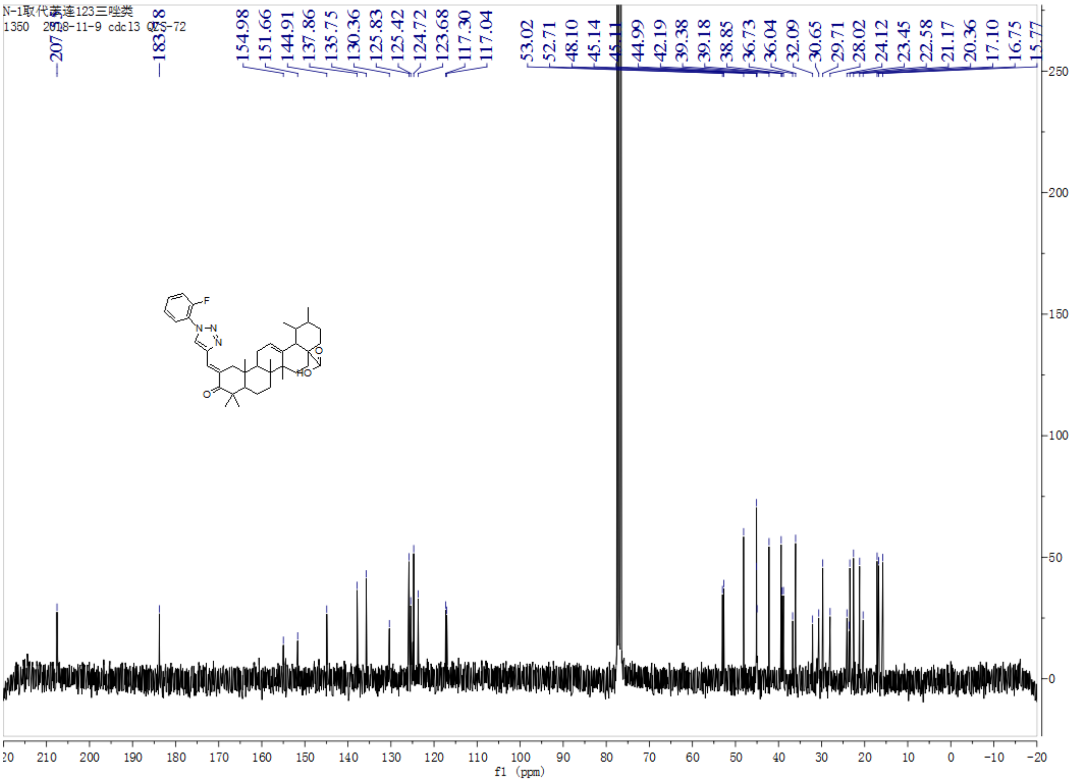

^{1}H NMR and ^{13}C NMR spectra of compound **1b**

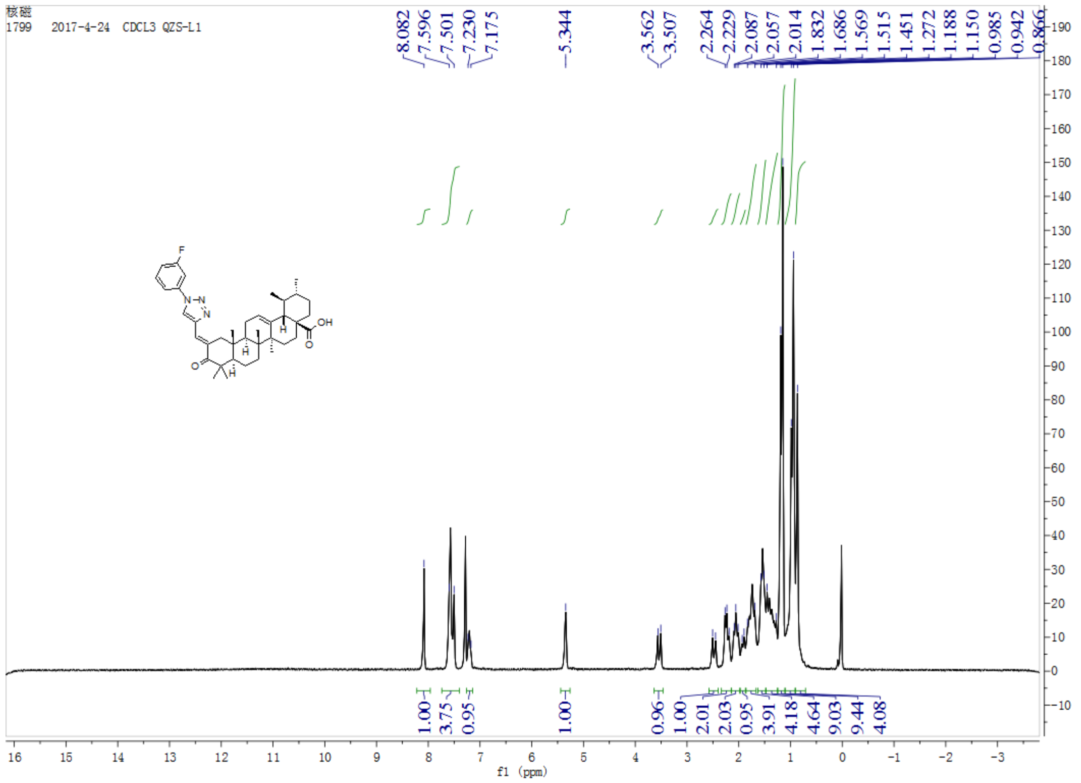

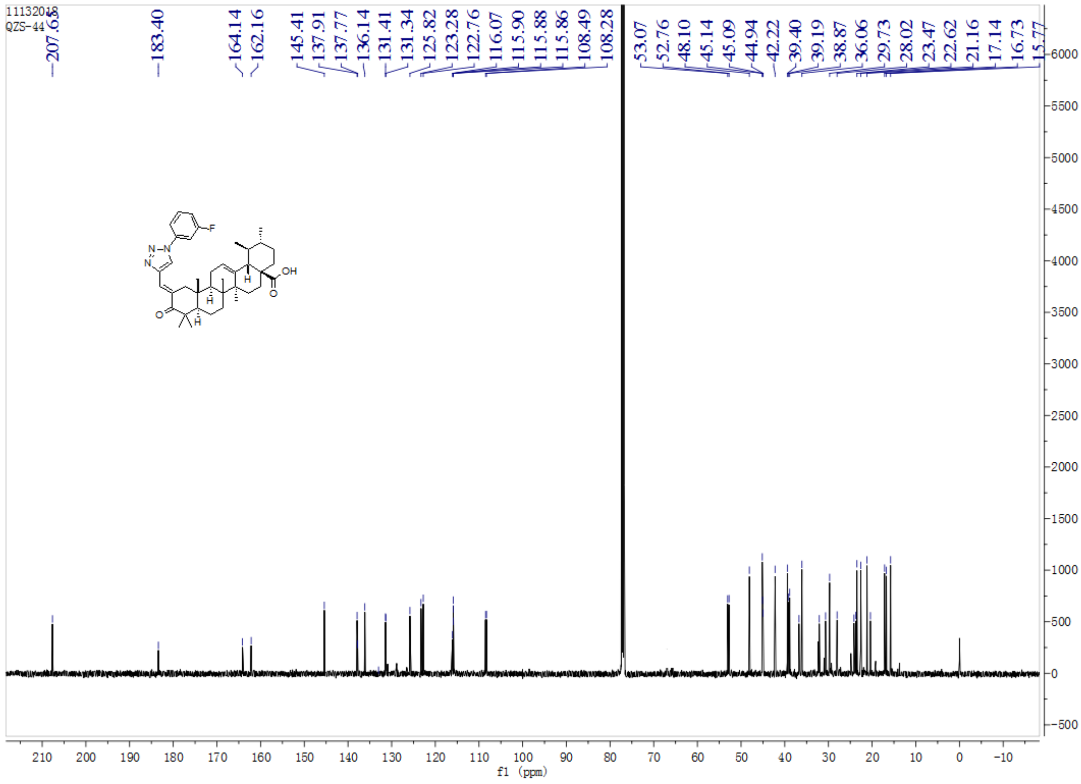

^{1}H NMR and ^{13}C NMR spectra of compound **1c**

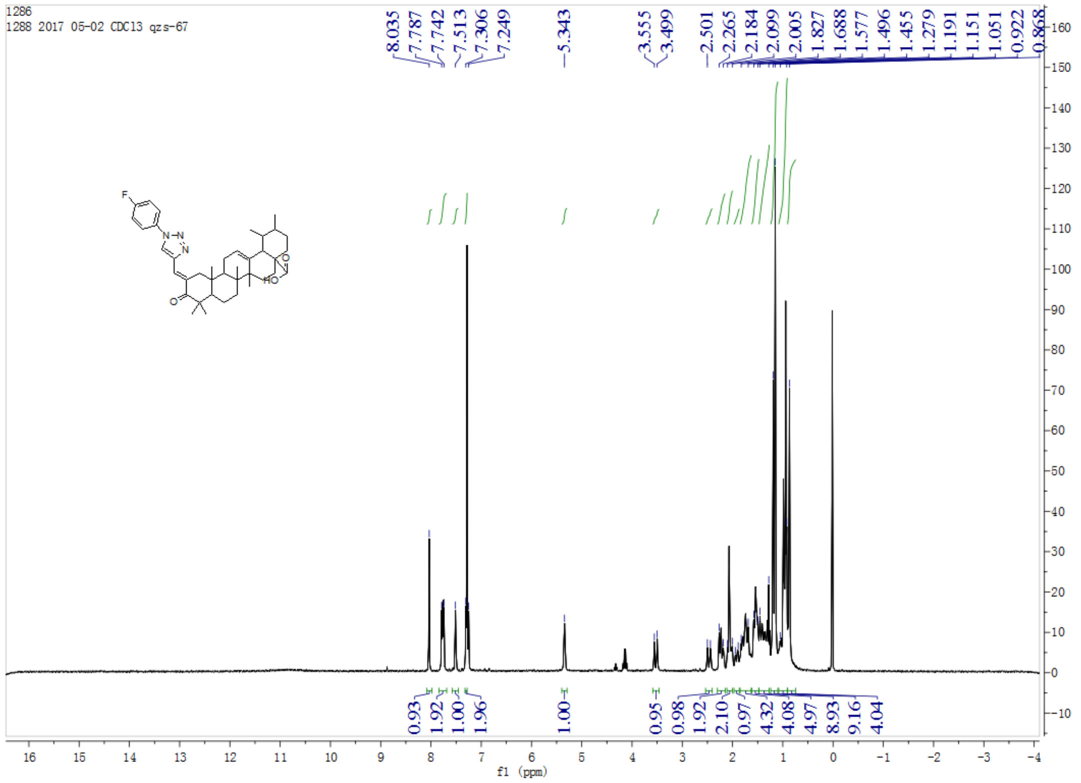

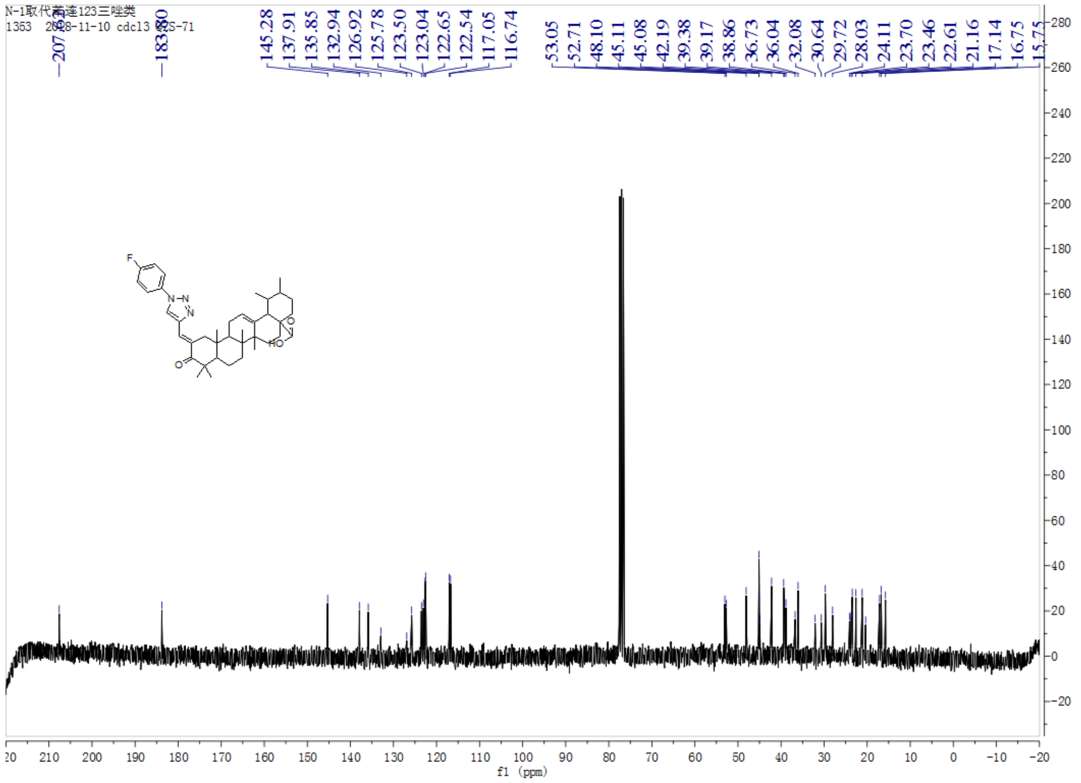

^{1}H NMR and ^{13}C NMR spectra of compound **1d**

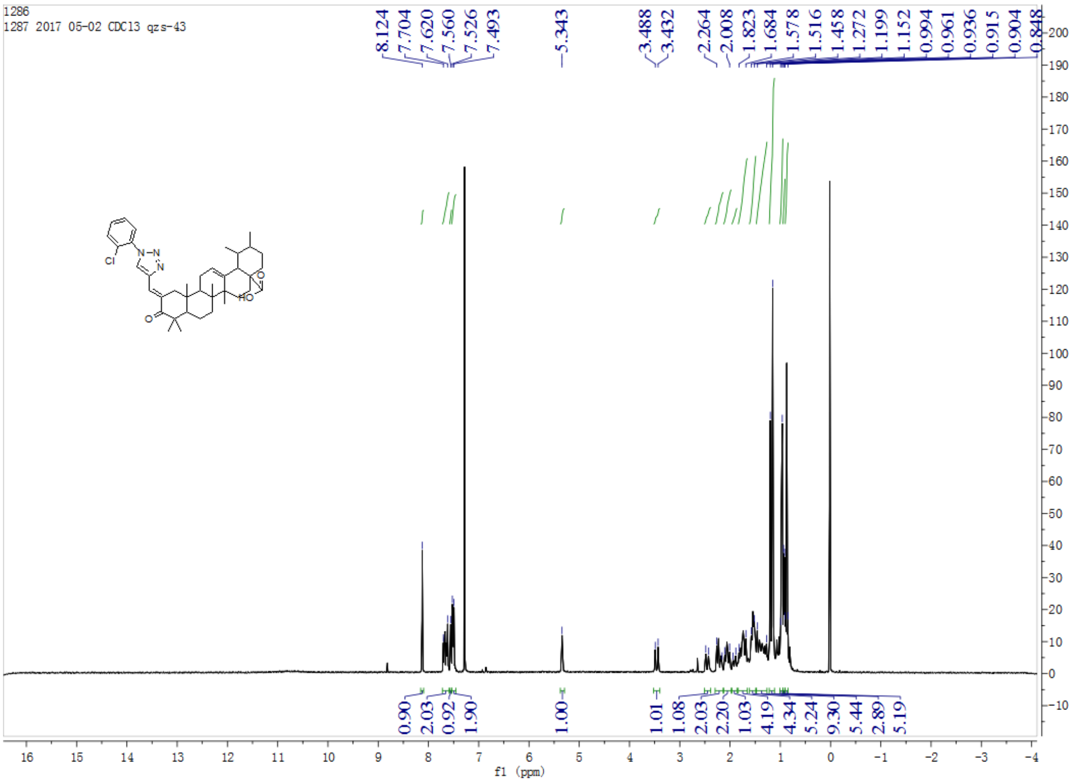

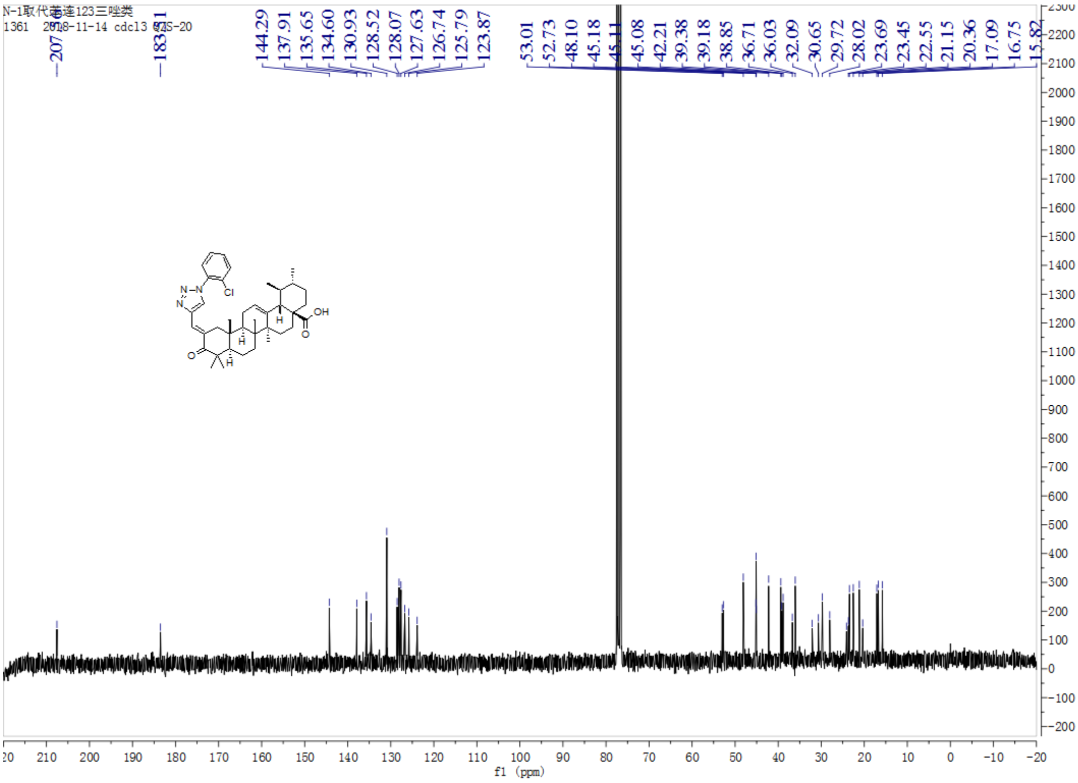

^{1}H NMR and ^{13}C NMR spectra of compound **1e**

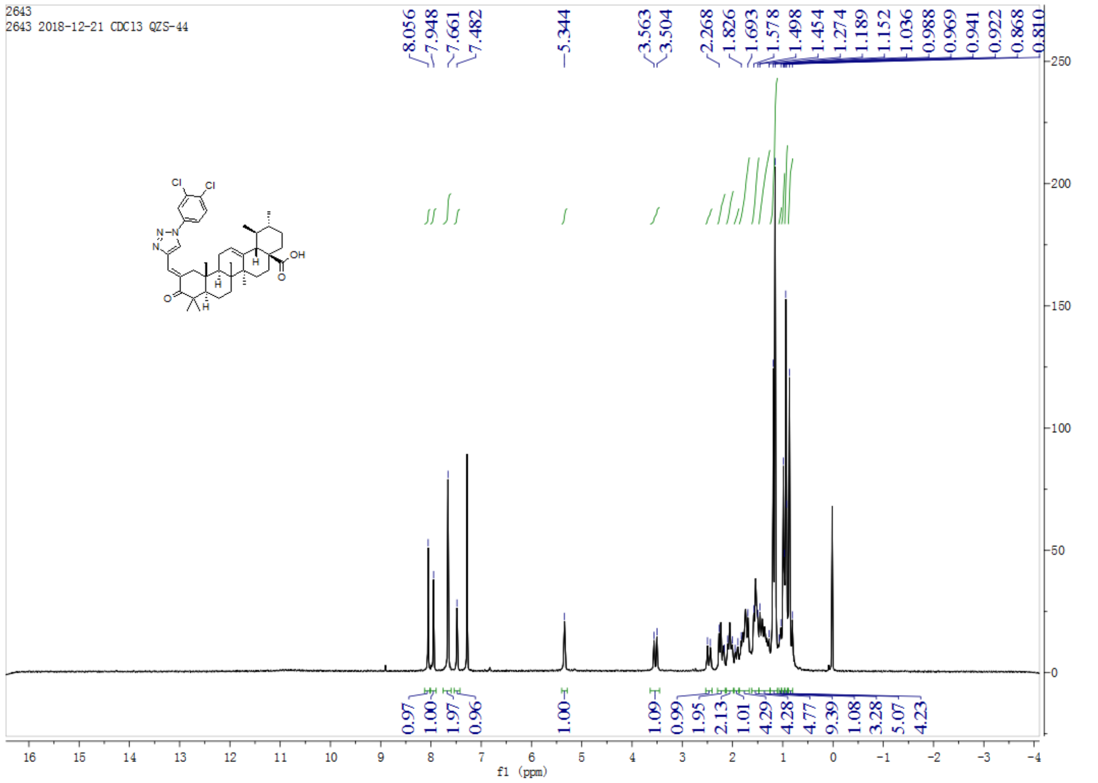

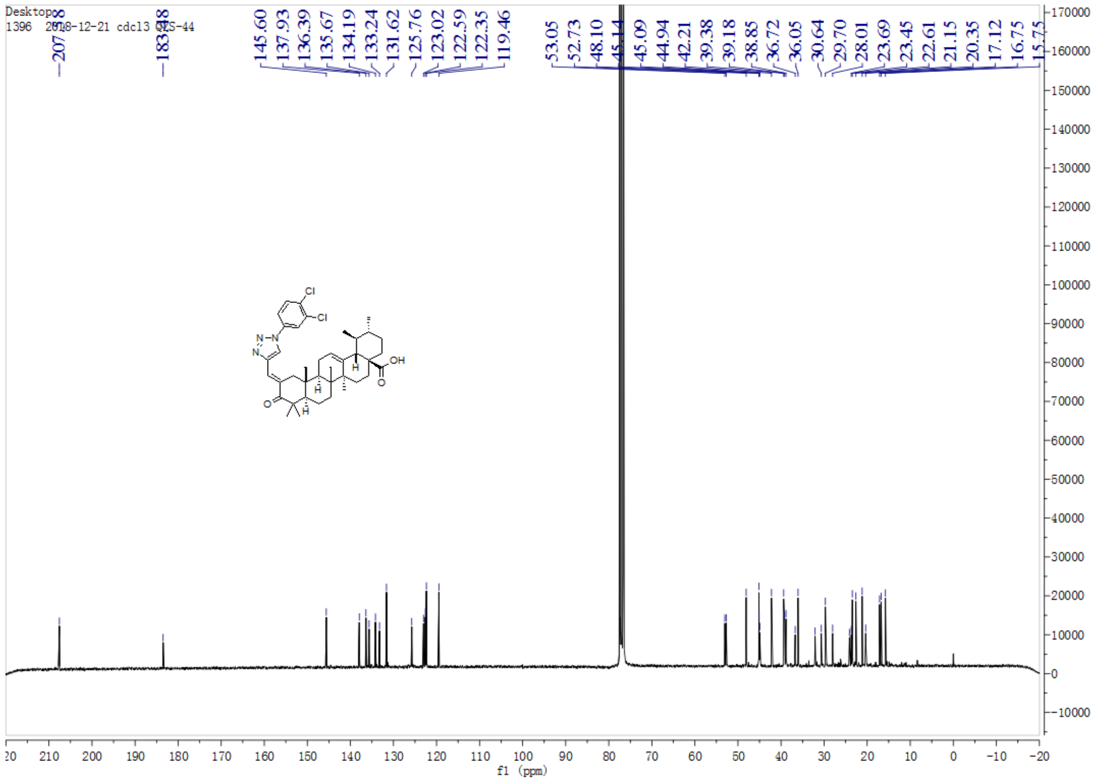

^{1}H NMR and ^{13}C NMR spectra of compound **1f**

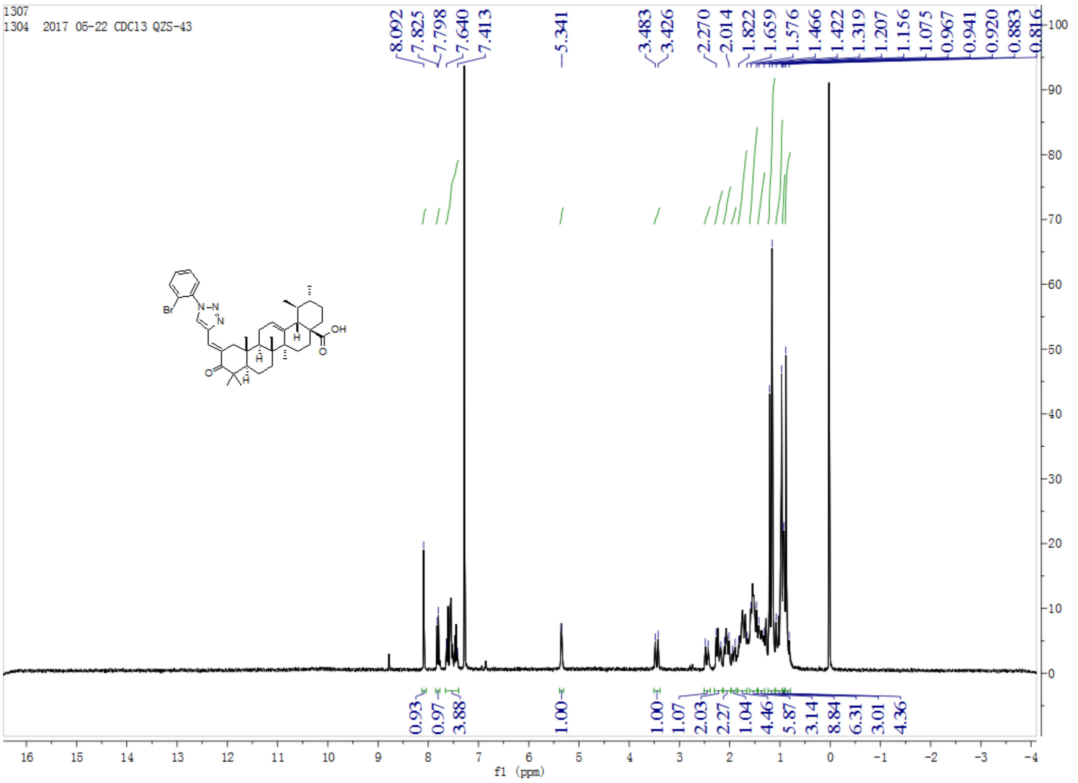

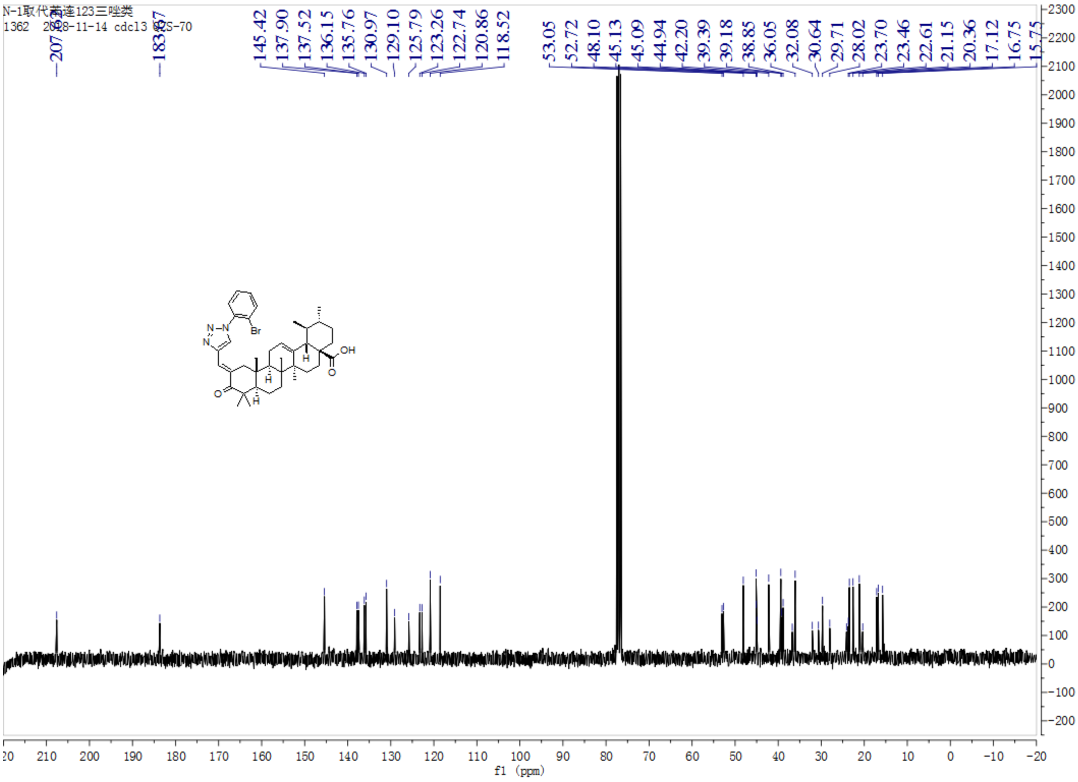

^{1}H NMR and ^{13}C NMR spectra of compound **1g**

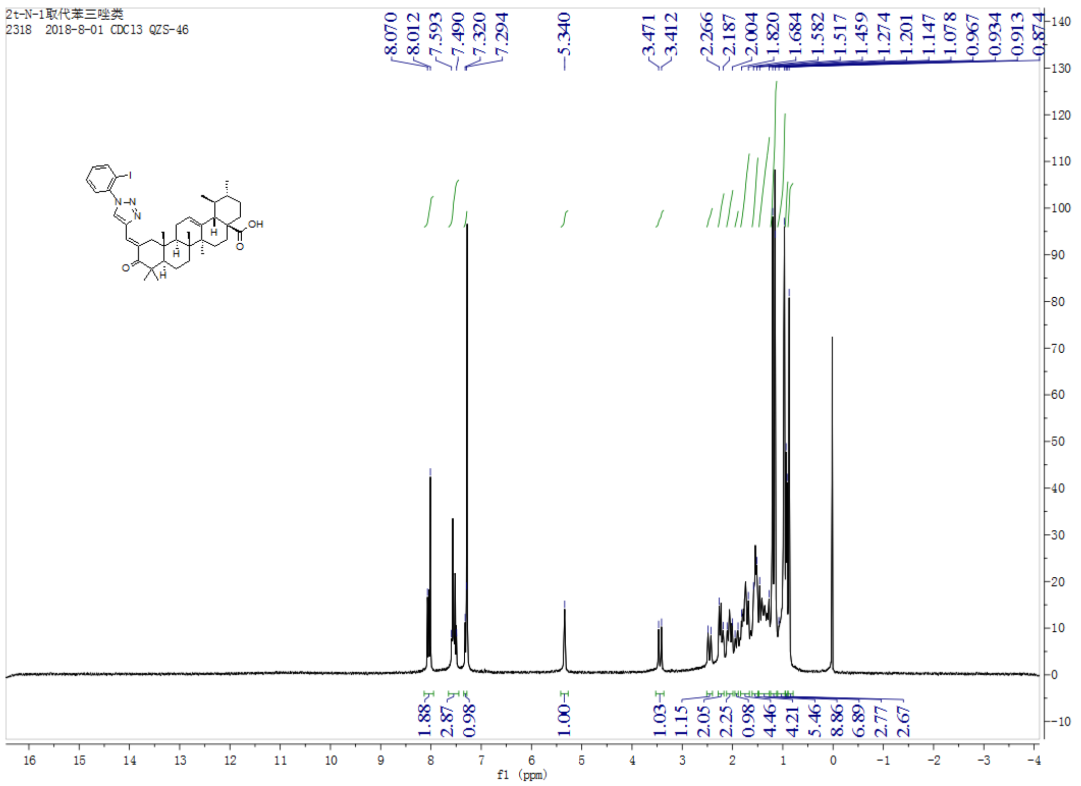

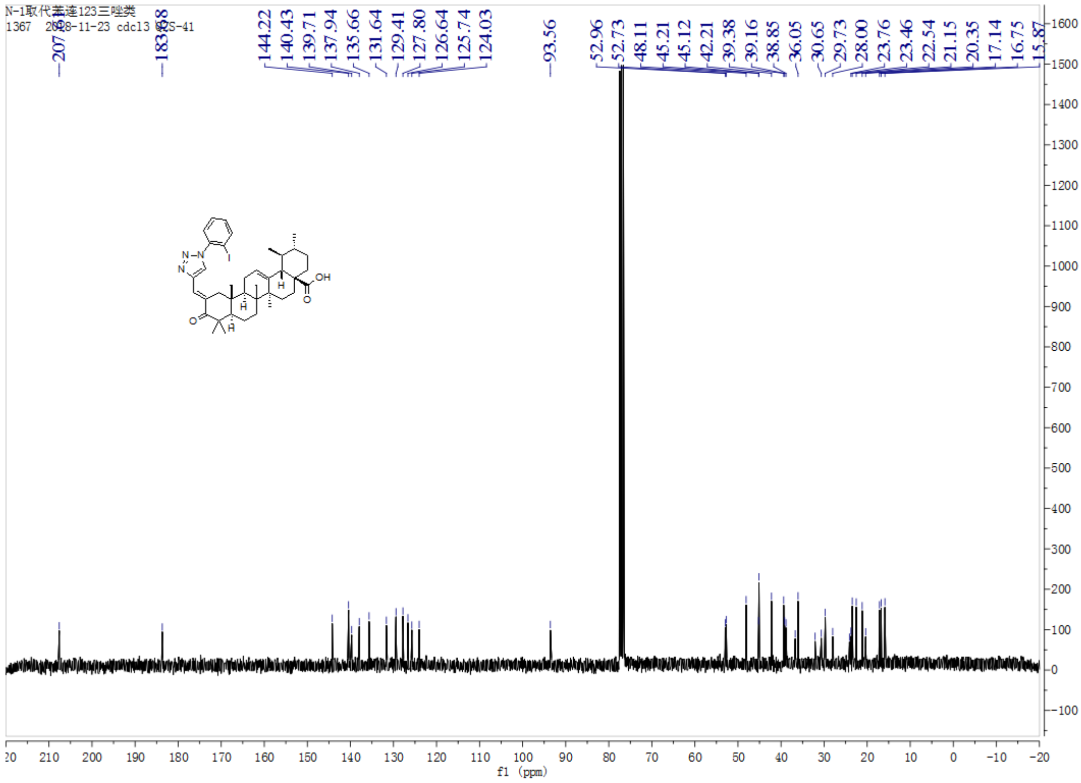

^{1}H NMR and ^{13}C NMR spectra of compound **1h**

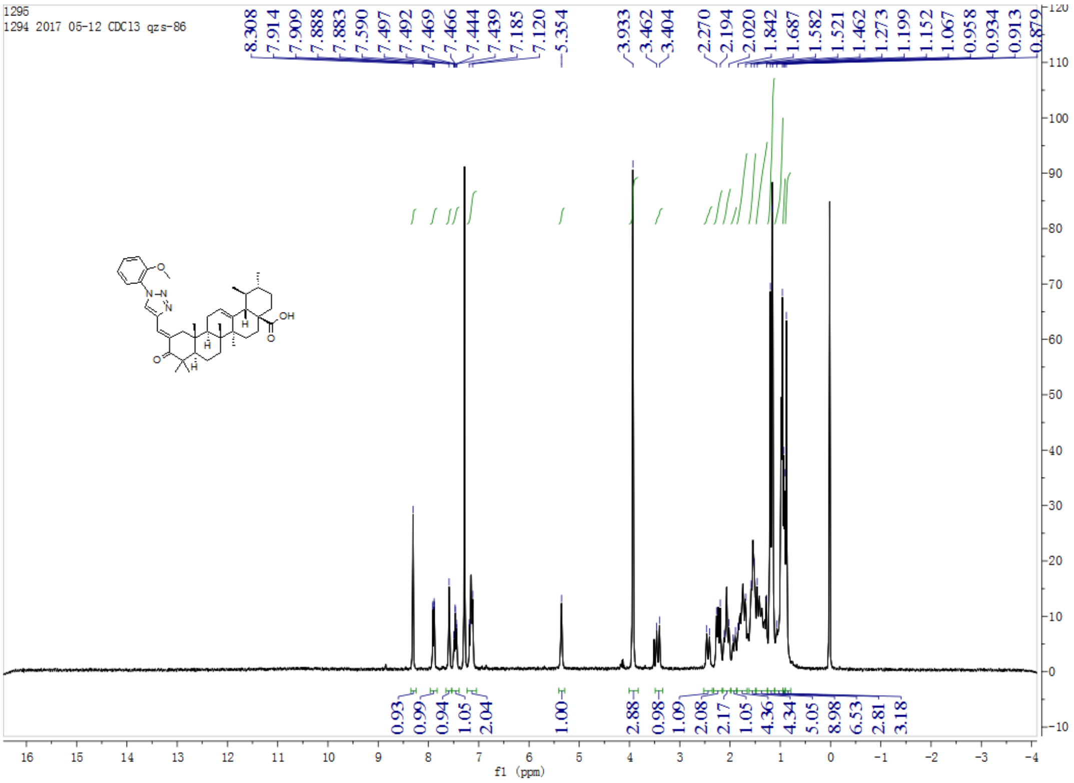

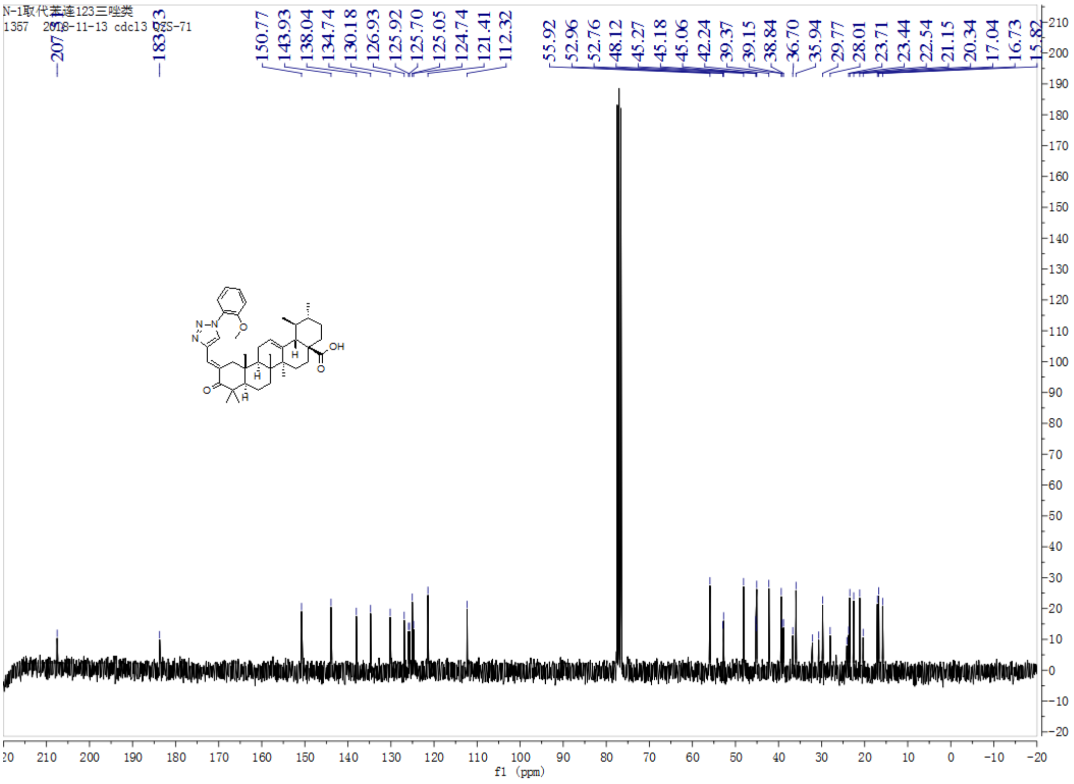

^{1}H NMR and ^{13}C NMR spectra of compound **1i**

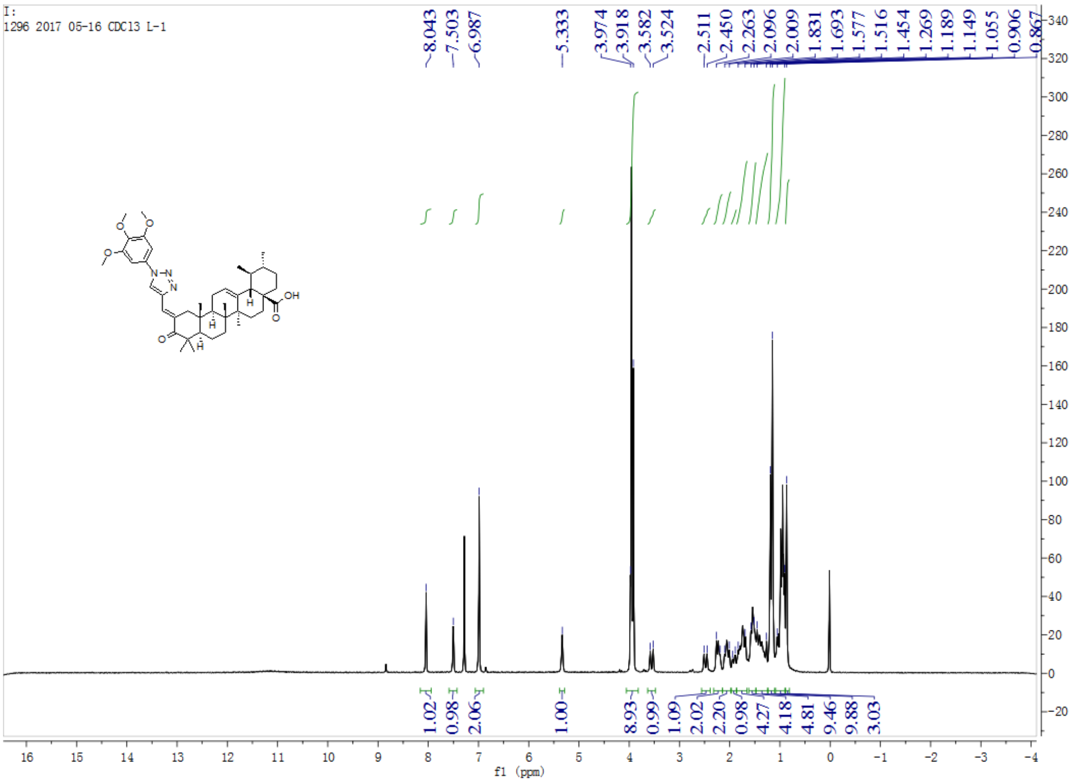

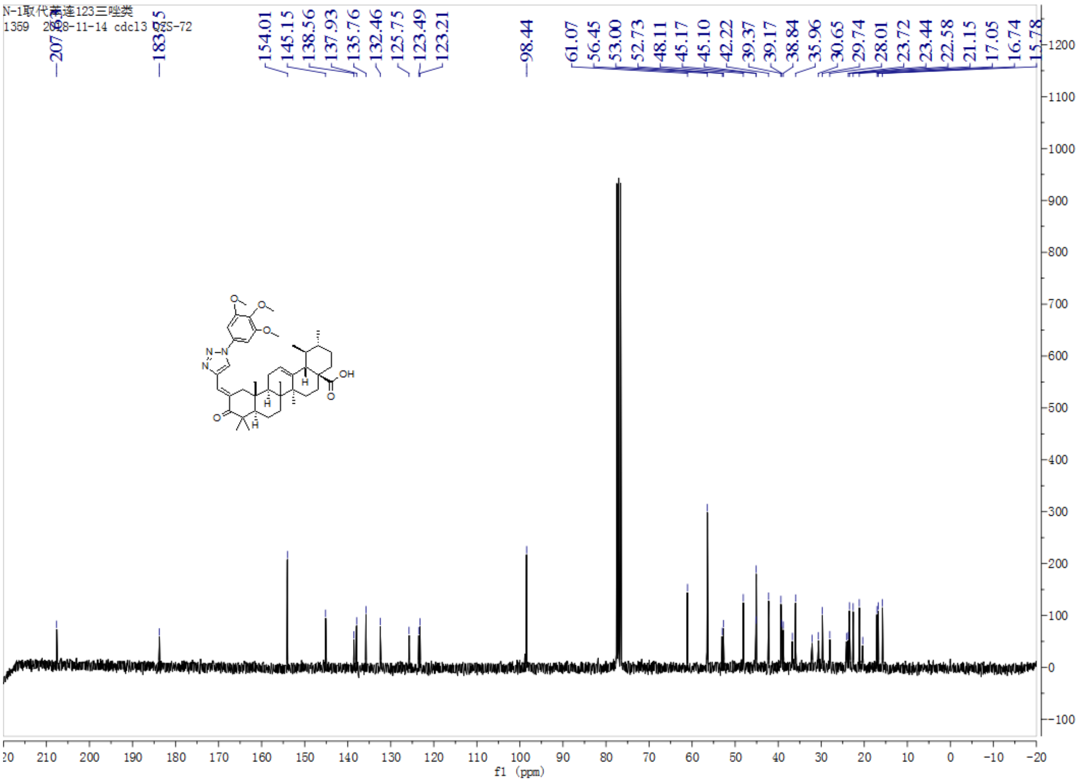

^{1}H NMR and ^{13}C NMR spectra of compound **1j**

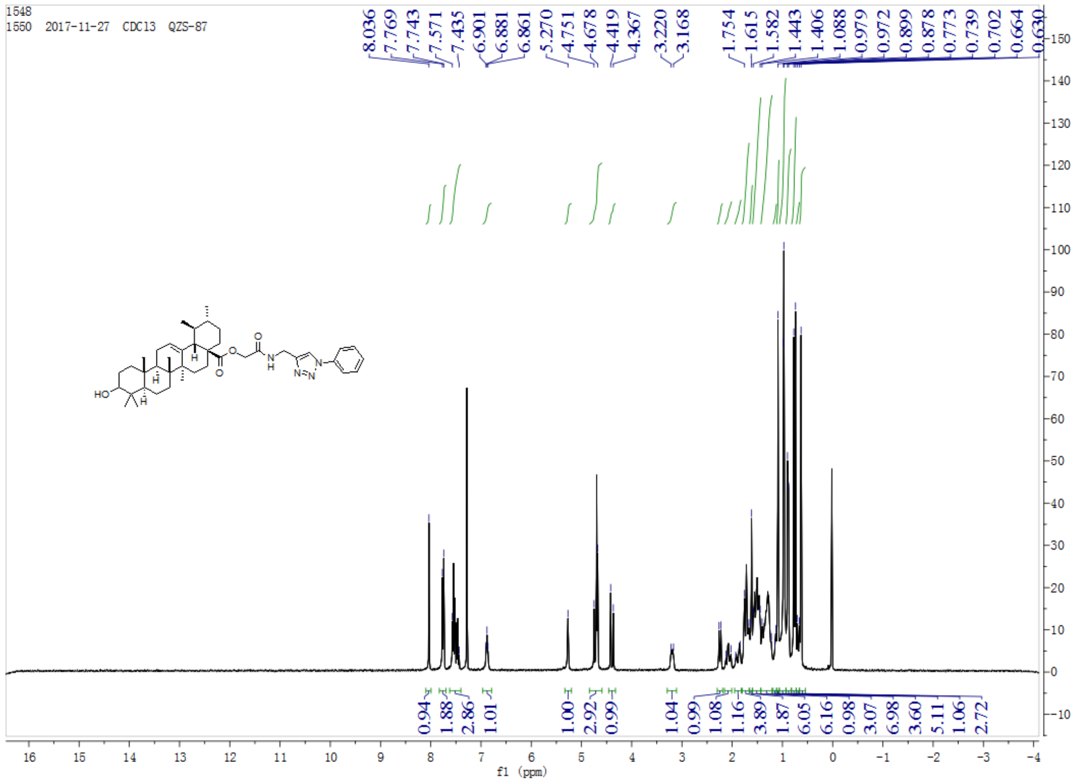

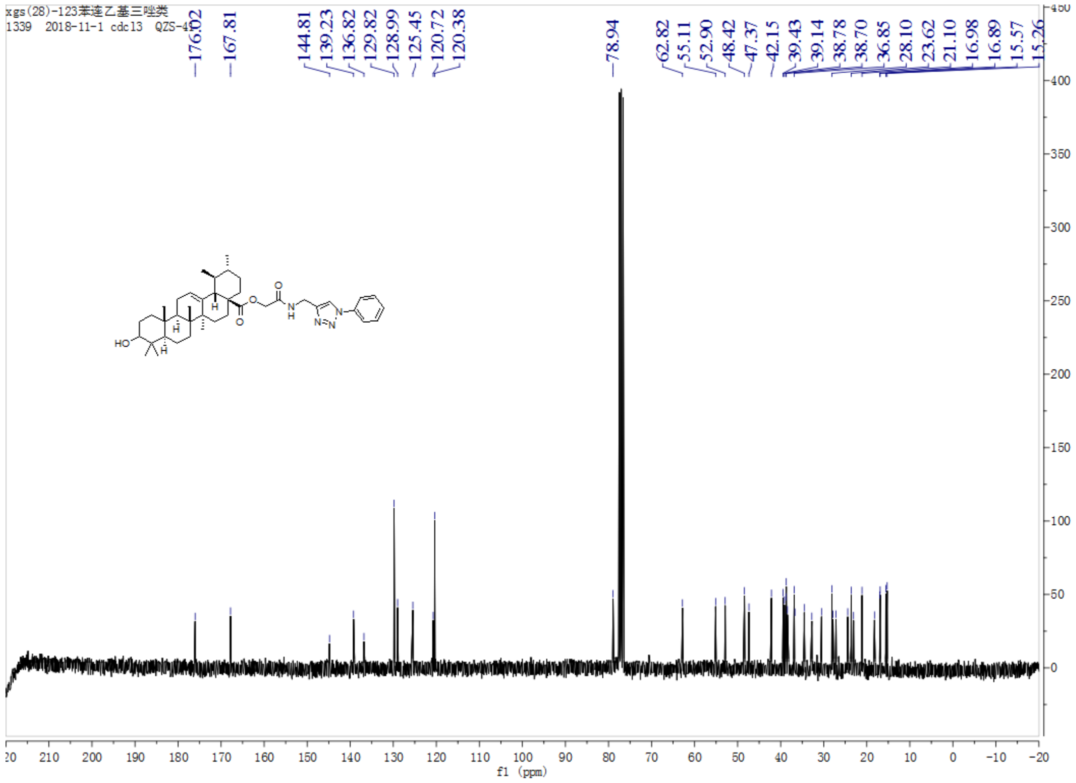

^{1}H NMR and ^{13}C NMR spectra of compound **2a**

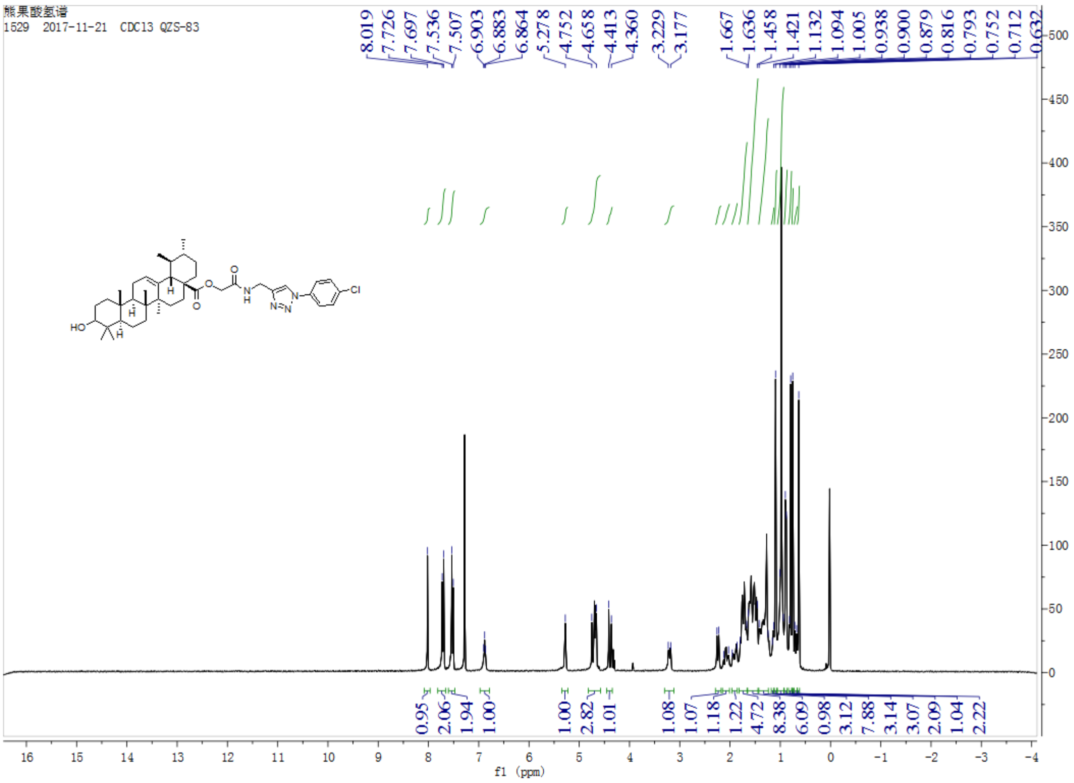

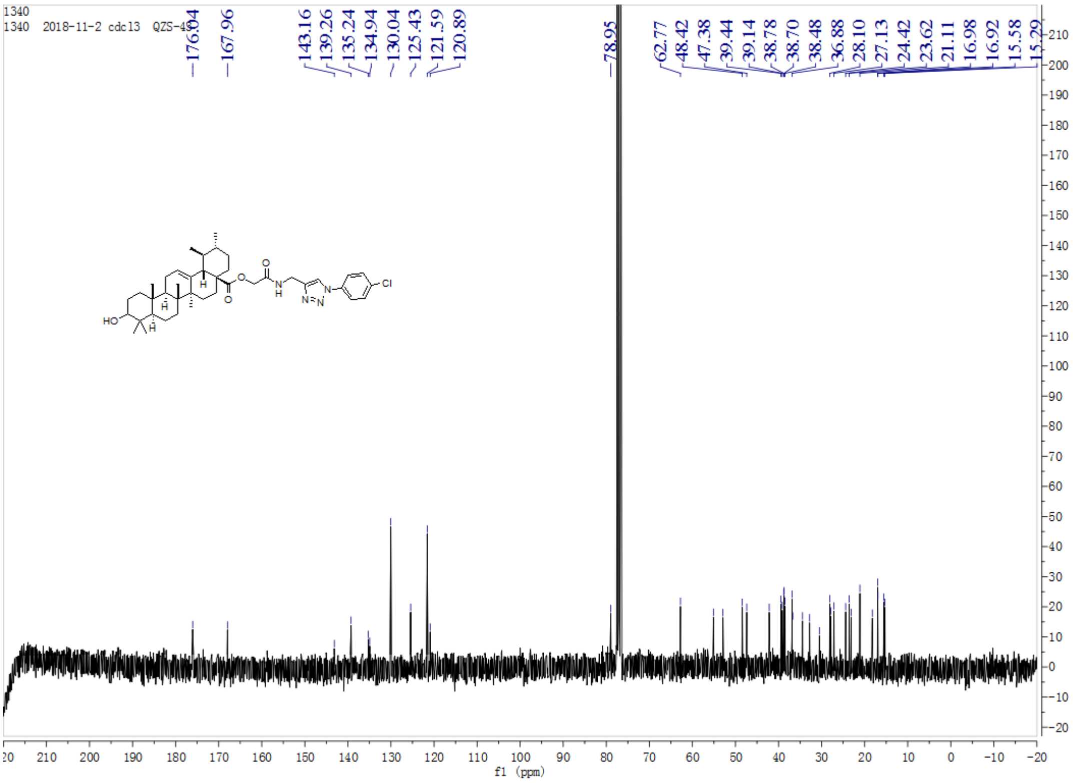

^{1}H NMR and ^{13}C NMR spectra of compound **2b**

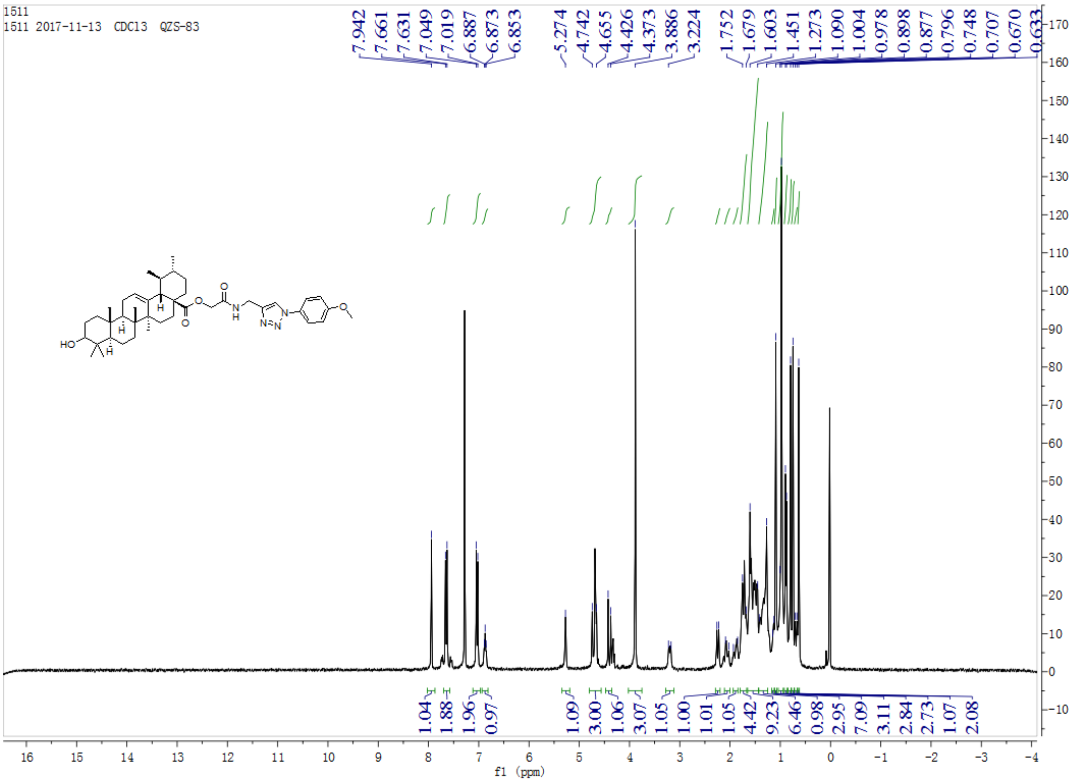

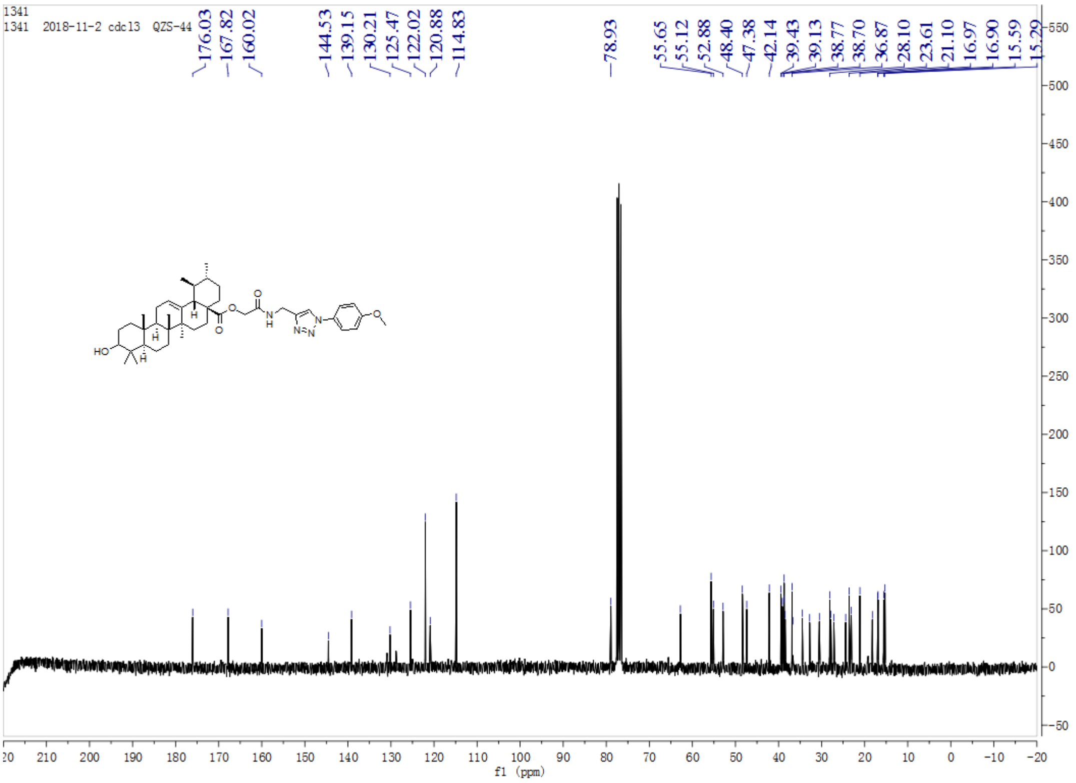

^{1}H NMR and ^{13}C NMR spectra of compound **2c**

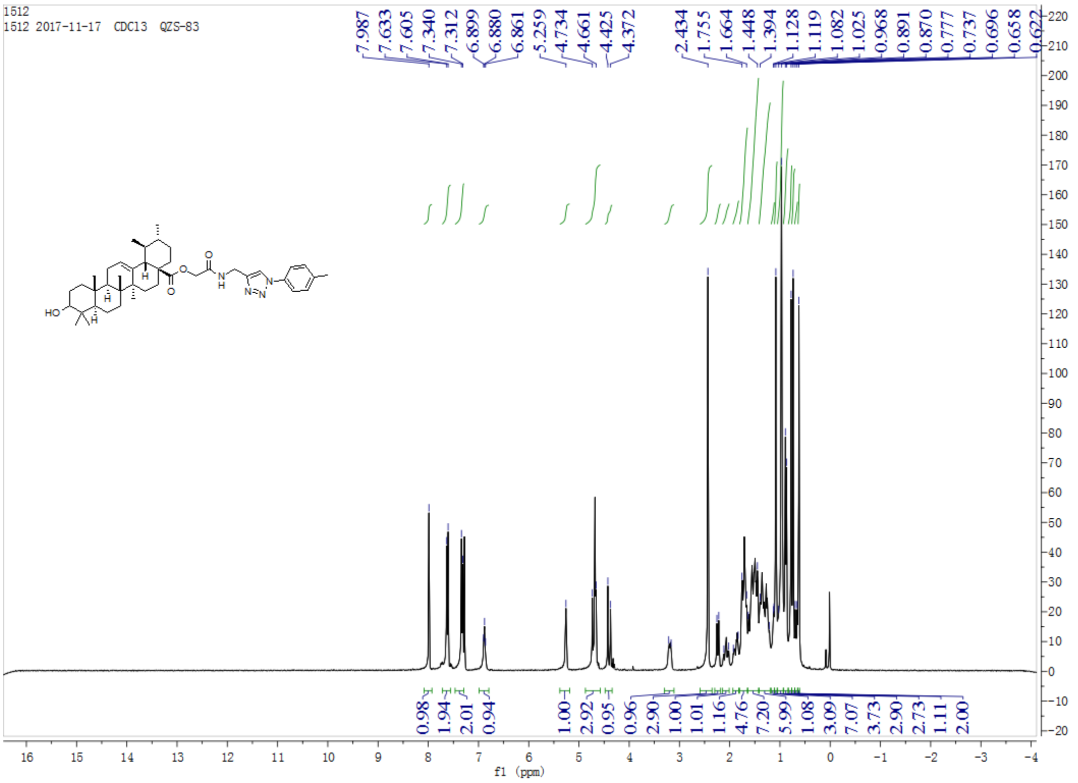

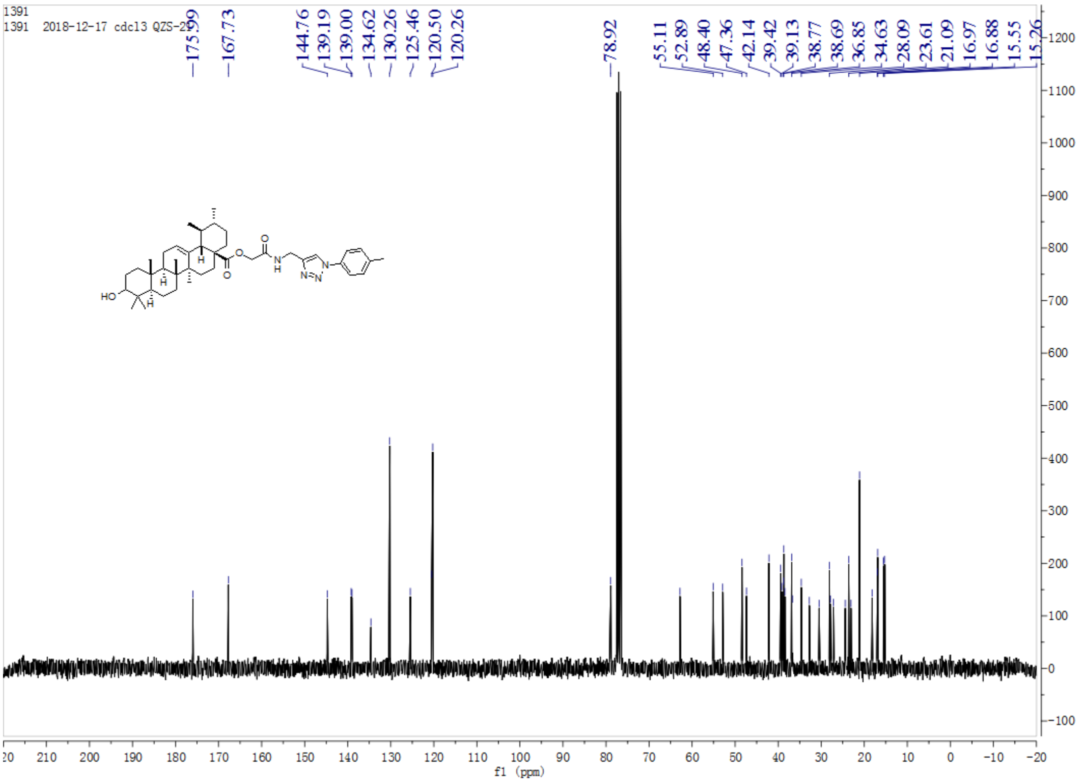

^{1}H NMR and ^{13}C NMR spectra of compound **2d**

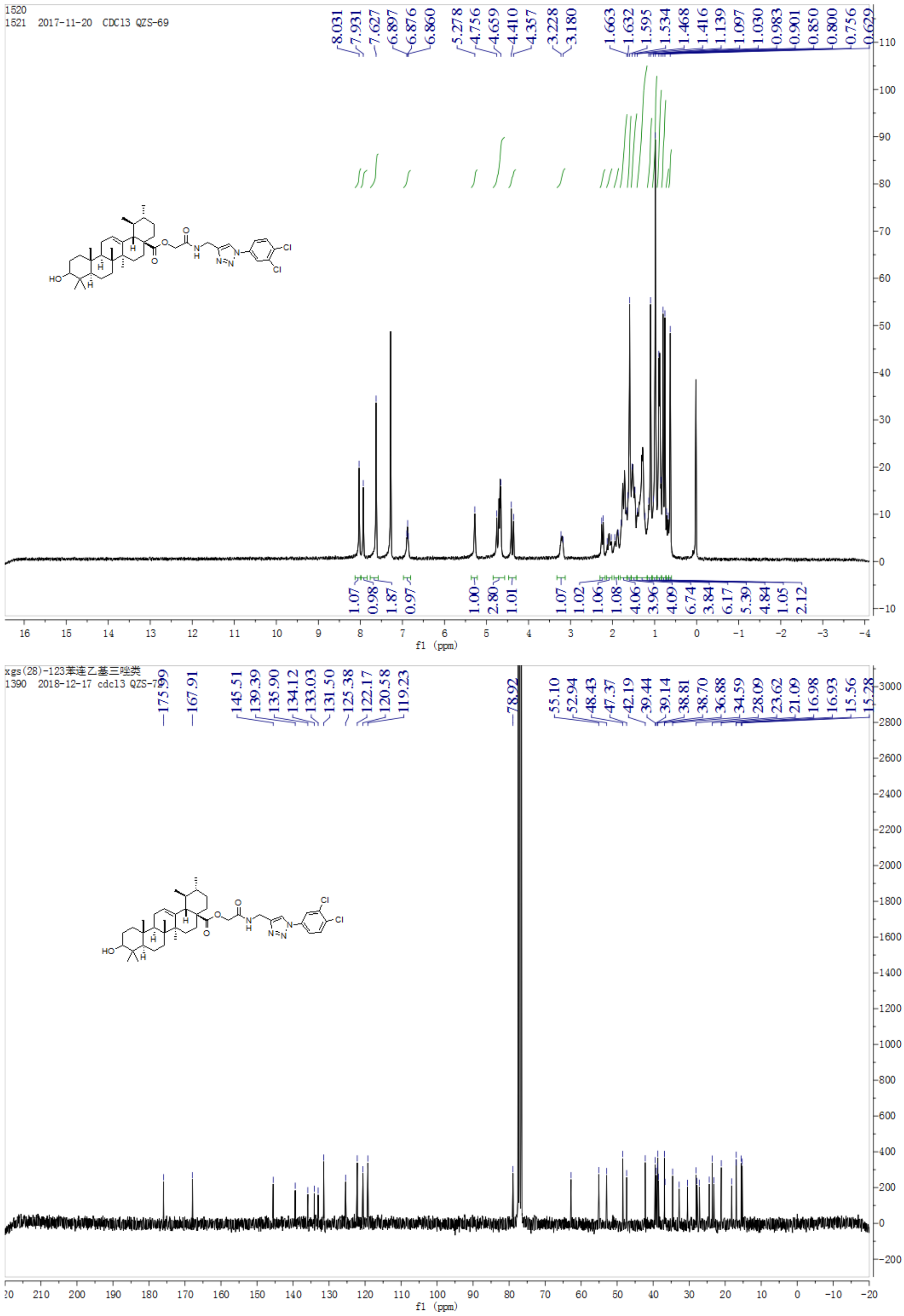

^{1}H NMR and ^{13}C NMR spectra of compound **2e**

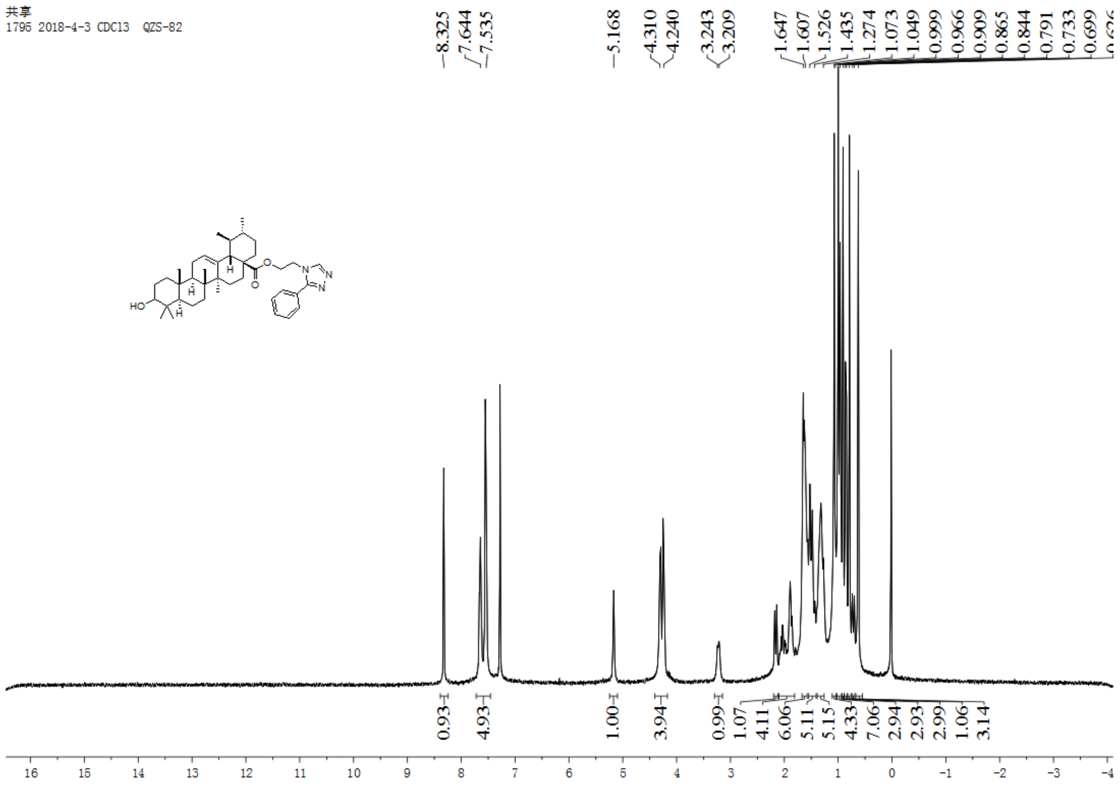

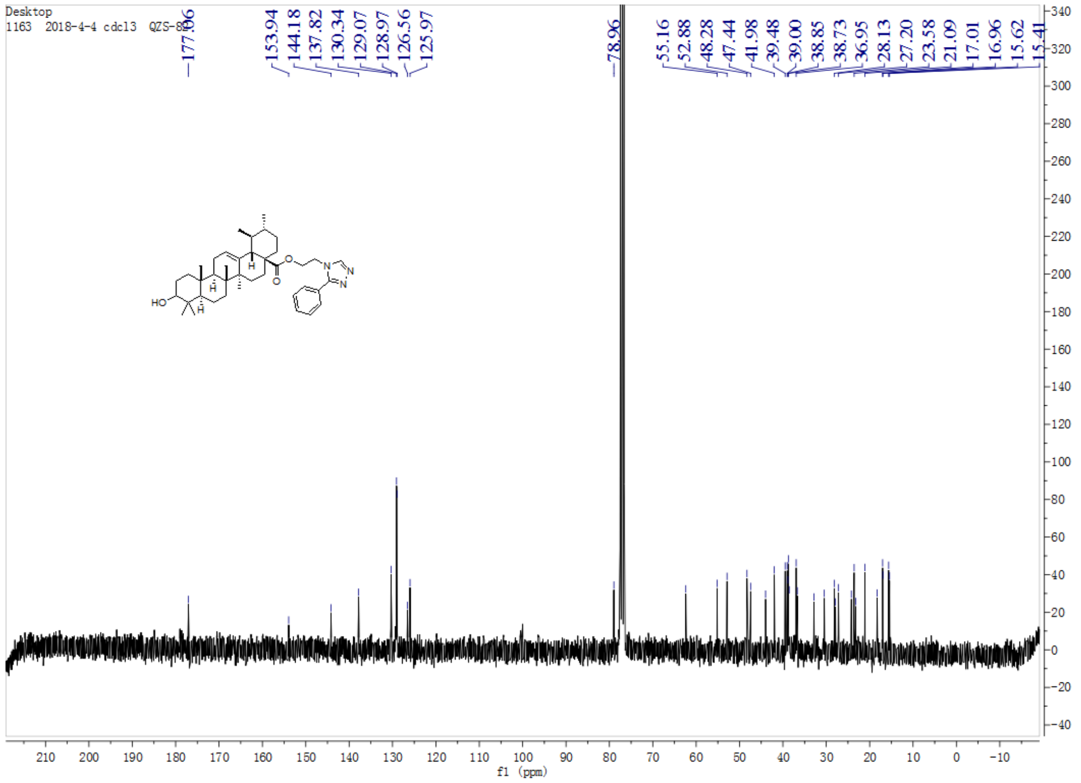

^{1}H NMR and ^{13}C NMR spectra of compound **3a**

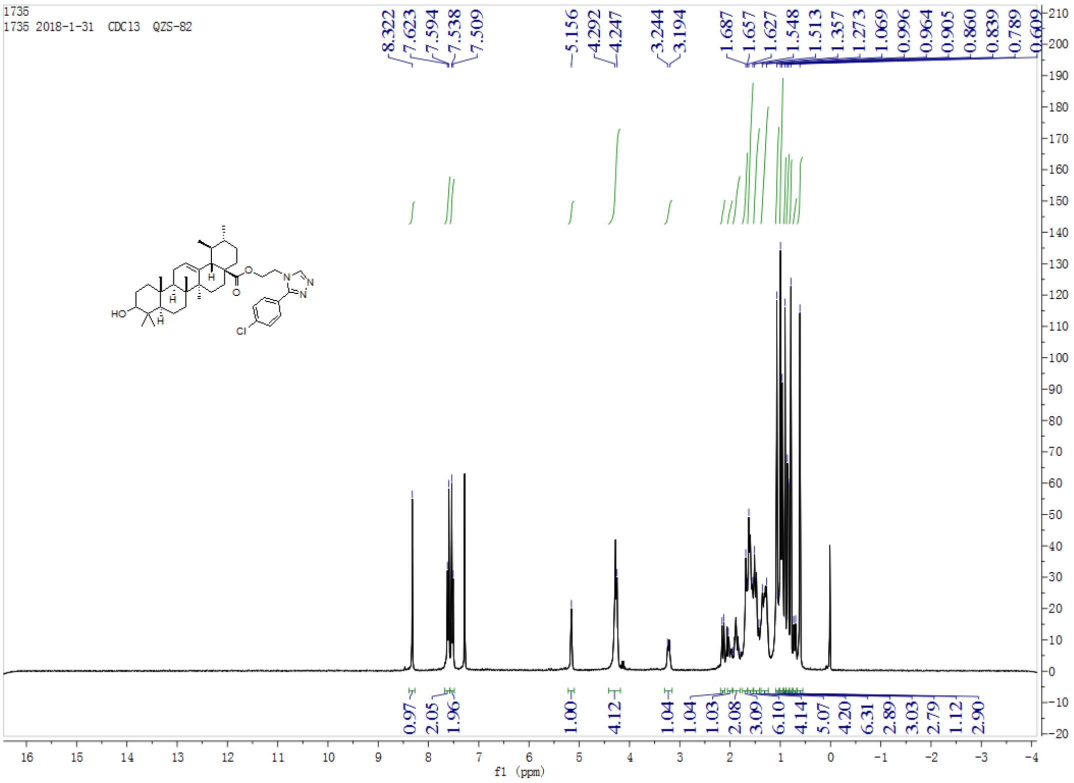

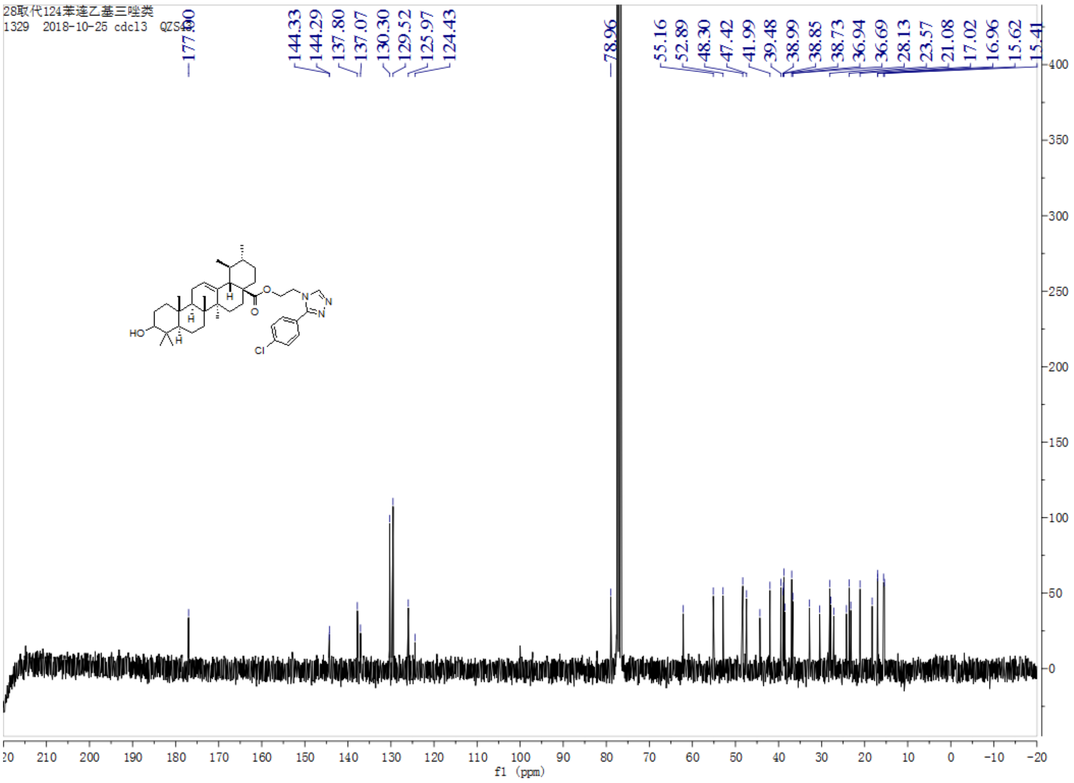

^{1}H NMR and ^{13}C NMR spectra of compound **3b**

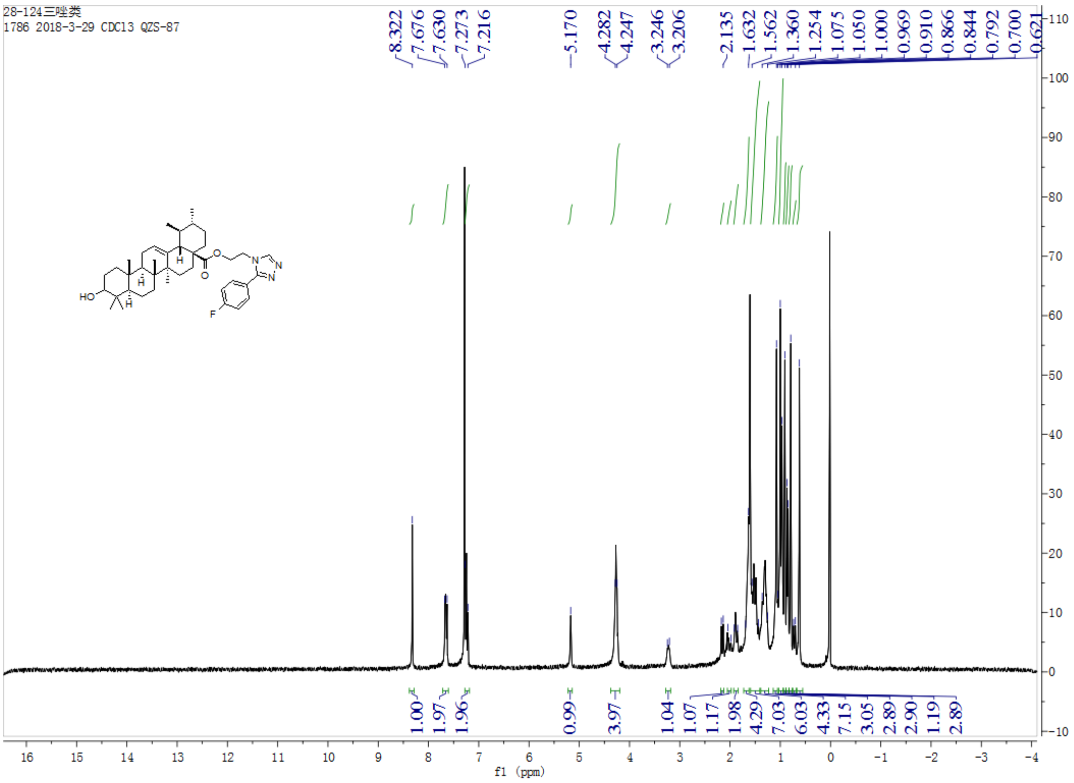

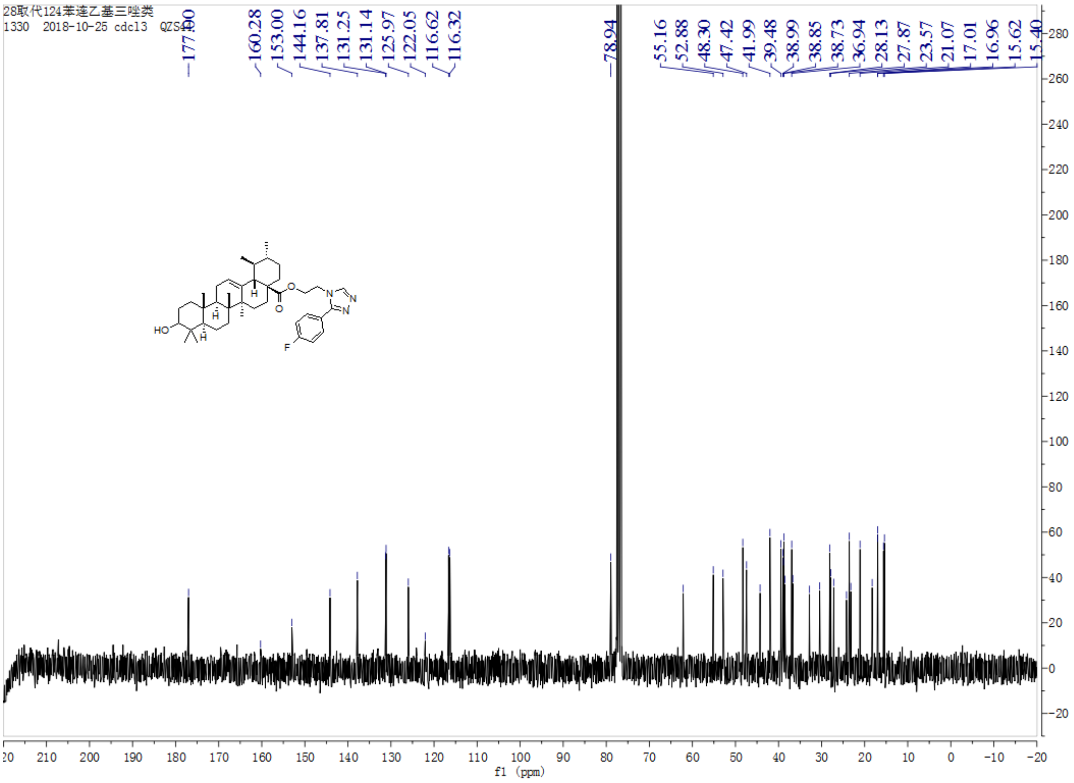

^{1}H NMR and ^{13}C NMR spectra of compound **3c**

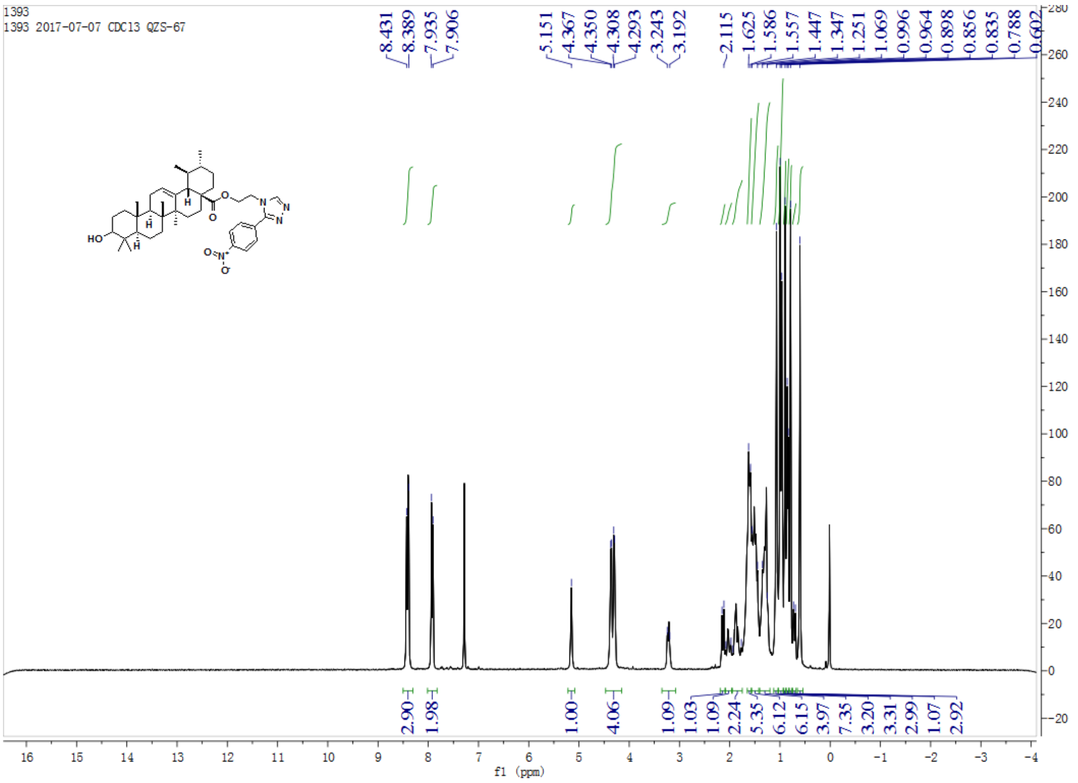

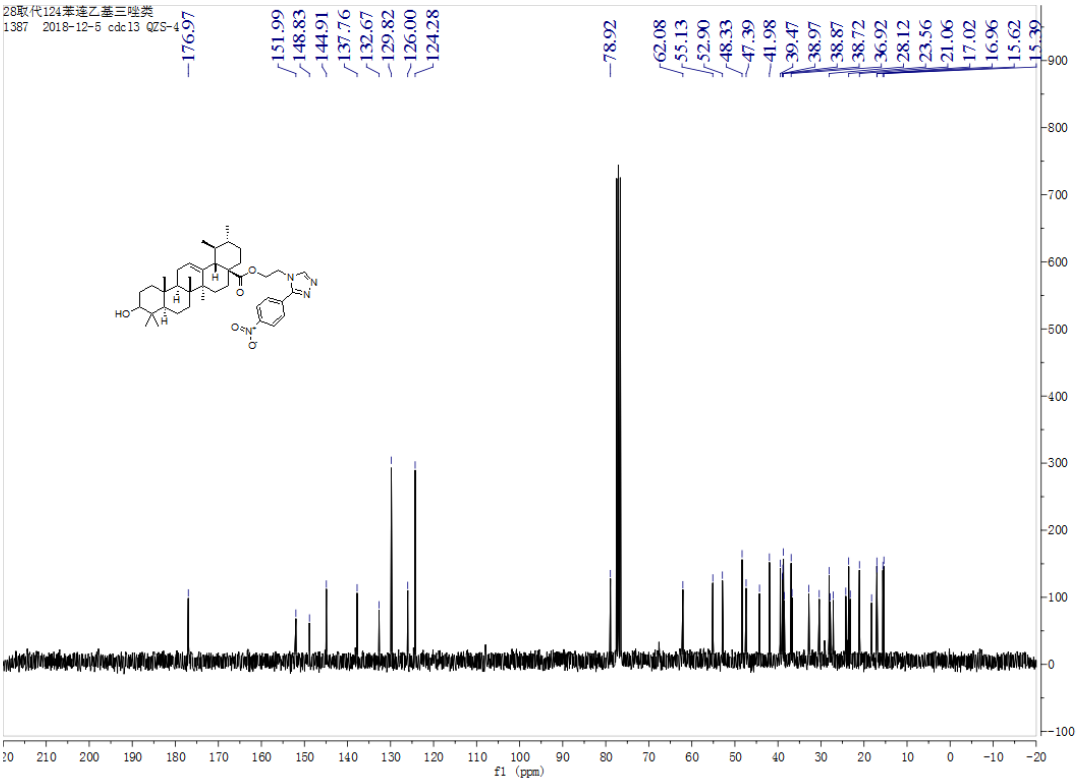

^{1}H NMR and ^{13}C NMR spectra of compound **3d**

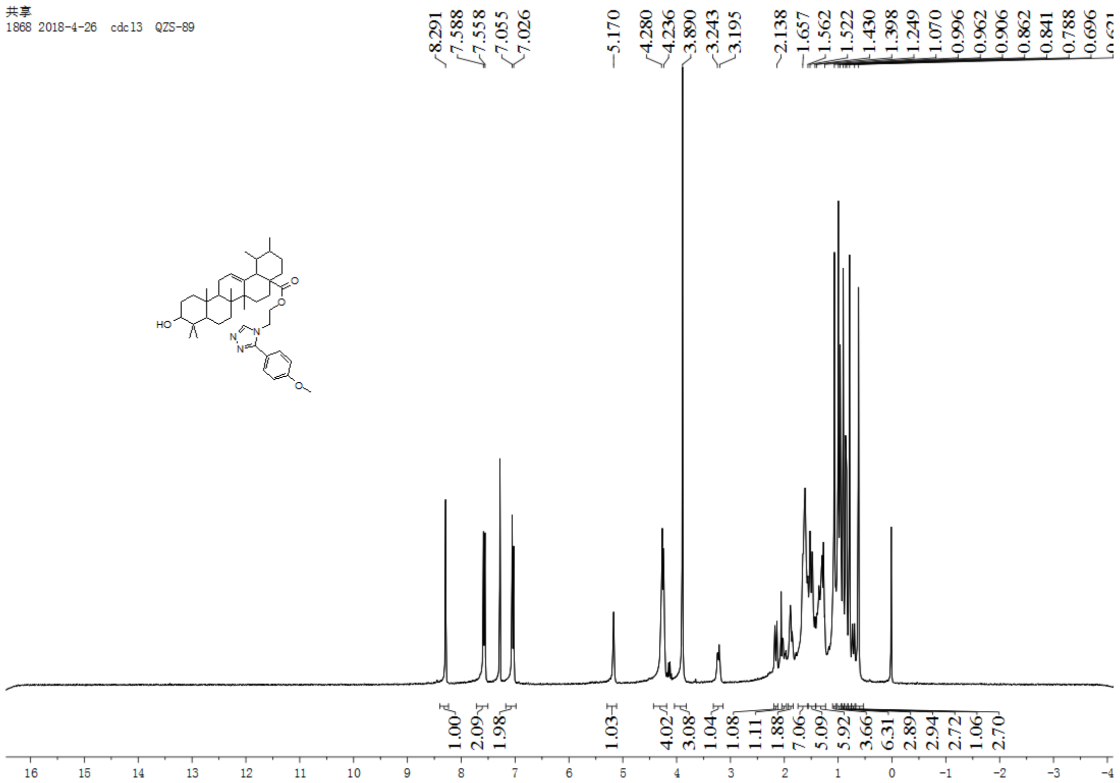

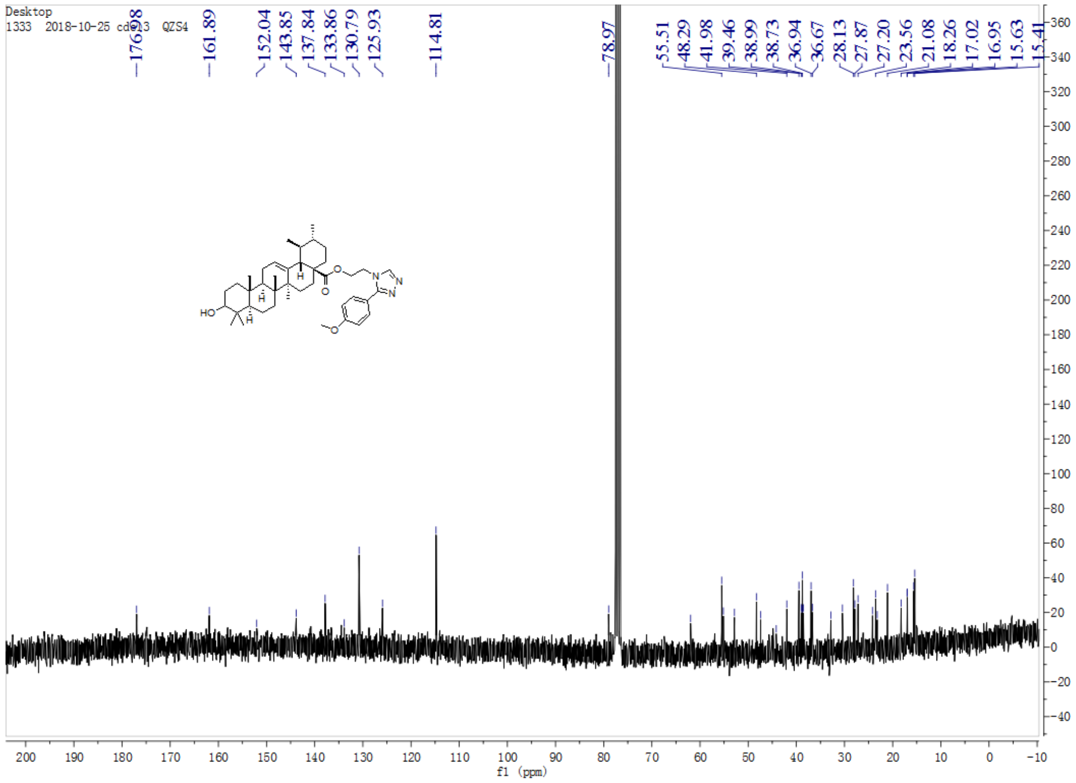

^{1}H NMR and ^{13}C NMR spectra of compound **3e**

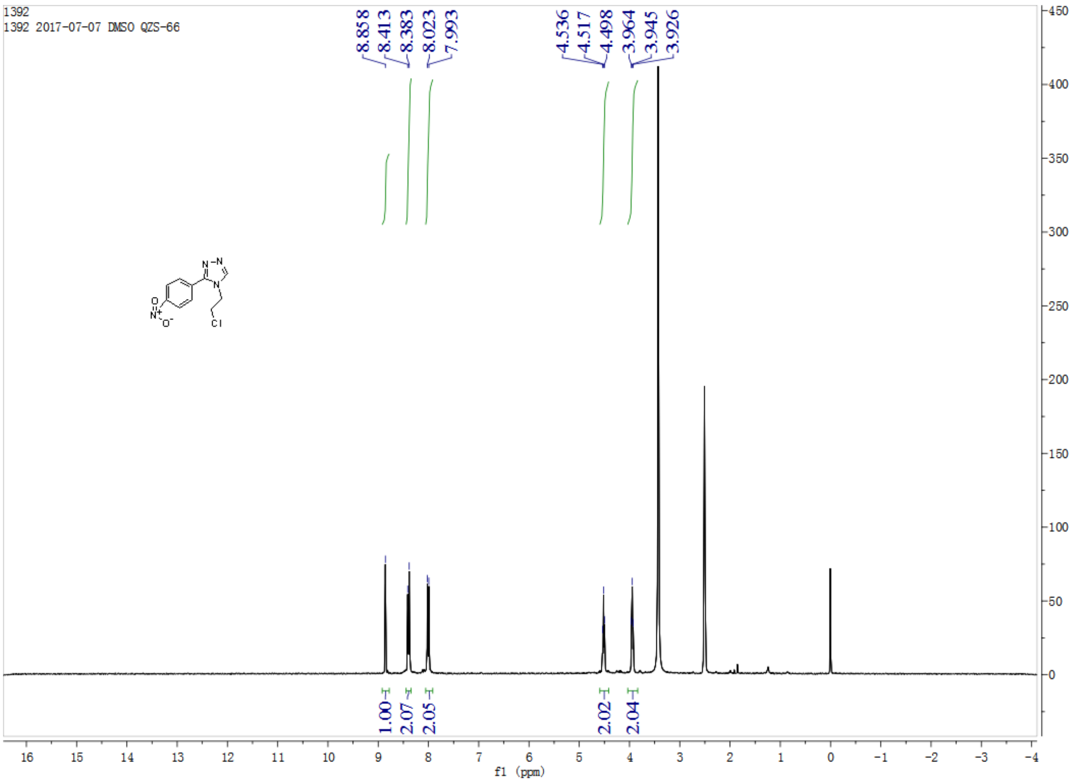

^{1}H-NMR spectra of intermediate **IIId**

^{1}H-NMR (DMSO-d_6, 300MHz, ppm): δ 8.86 (s, 1H, triazole-H), 8.40 (d, *J* = 9.0 Hz, 2H, Ar-H), 8.10 (d, *J* = 9.0 Hz, 2H, Ar-H), 4.52 (t, *J*= 5.7 Hz, 2H, -C<u>H</u>$_2$-Cl), 3.95 (t, *J*= 5.7 Hz, 2H, N-C<u>H</u>$_2$-).
